# Nernst-Planck-Gaussian modelling of electrodiffusional recovery from ephaptic excitation between mammalian cardiomyocytes

**DOI:** 10.3389/fphys.2023.1280151

**Published:** 2024-01-03

**Authors:** Joshua A. Morris, Oliver J. Bardsley, Samantha C. Salvage, Antony P. Jackson, Hugh R. Matthews, Christopher L-H. Huang

**Affiliations:** ^1^ Physiological Laboratory, University of Cambridge, Cambridge, United Kingdom; ^2^ Department of Veterinary Medicine, University of Cambridge, Cambridge, United Kingdom; ^3^ Department of Biochemistry, University of Cambridge, Cambridge, United Kingdom

**Keywords:** ephaptic conduction, cardiomyocytes, action potential propagation, sodium channels, electrodiffusion

## Abstract

**Introduction:** In addition to gap junction conduction, recent reports implicate possible ephaptic coupling contributions to action potential (AP) propagation between successive adjacent cardiomyocytes. Here, AP generation in an active cell, withdraws Na^+^ from, creating a negative potential within, ephaptic spaces between the participating membranes, *activating* the initially quiescent neighbouring cardiomyocyte. However, sustainable ephaptic transmission requires subsequent complete *recovery* of the ephaptic charge difference. We explore physical contributions of passive electrodiffusive ion exchange with the remaining extracellular space to this recovery for the first time.

**Materials and Methods:** Computational, finite element, analysis examined limiting, temporal and spatial, ephaptic [Na^+^], [Cl^−^], and the consequent Gaussian charge differences and membrane potential recovery patterns following a Δ*V*∼130 mV AP upstroke at physiological (37°C) temperatures. This incorporated Nernst-Planck formalisms into equations for the time-dependent spatial concentration gradient profiles.

**Results:** Mammalian atrial, ventricular and purkinje cardiomyocyte ephaptic junctions were modelled by closely apposed circularly symmetric membranes, specific capacitance 1 μF cm^-2^, experimentally reported radii *a =* 8,000, 12,000 and 40,000 nm respectively and ephaptic axial distance *w* = 20 nm. This enclosed an ephaptic space containing principal ions initially at normal extracellular [Na^+^] = 153.1 mM and [Cl^−^] = 145.8 mM, respective diffusion coefficients *D*_Na_ = 1.3 × 10^9^ and *D*_Cl_ = 2 × 10^9^ nm^2^s^-1^. Stable, concordant computational solutions were confirmed exploring ≤1,600 nm mesh sizes and Δ*t*≤0.08 ms stepsize intervals. The corresponding membrane voltage profile changes across the initially quiescent membrane were obtainable from computed, graphically represented *a* and *w*-dependent ionic concentration differences adapting Gauss’s flux theorem. Further simulations explored biological variations in ephaptic dimensions, membrane anatomy, and diffusion restrictions within the ephaptic space. Atrial, ventricular and Purkinje cardiomyocytes gave 40, 180 and 2000 ms 99.9% recovery times, with 720 or 360 ms high limits from doubling ventricular radius or halving diffusion coefficient. Varying *a*, and *D*_Na_ and *D*_Cl_ markedly affected recovery time-courses with logarithmic and double-logarithmic relationships, Varying *w* exerted minimal effects.

**Conclusion:** We thereby characterise the properties of, and through comparing atrial, ventricular and purkinje recovery times with interspecies *in vivo* background cardiac cycle duration data, (blue whale ∼2000, human∼90, Etruscan shrew, ∼40 ms) can determine physical limits to, electrodiffusive contributions to ephaptic recovery.

## 1 Introduction

### 1.1 Gap junctional and ephaptic conduction of cardiomyocyte excitation

It has long been established that action potential (AP) conduction between successive cardiomyocytes within myocardial syncytia involves local circuit current flow through low resistance gap junctions (GJs) (Rohr, 2004). The required intercellular conductivities largely arise from connexin Cx40 (unit conductances 120–180 pS) and/or Cx43 channels (unit conductances 40–70 or 90-100 pS depending on phosphorylation conditions) bridging adjoining cells (Moreno et al., 1994; Traub et al., 1994). Ventricular myocyte gap junctions immunostained with anti-Cx43 (and anti-Cx45), but not anti-Cx40 antibodies. Atrial gap junctions immunostained with both anti-Cx40 and anti-Cx43 (and anti-Cx45) antibodies. Double whole cell voltage clamped rabbit atrial and ventricular gap junctions conductance nevertheless showed similar respective, 169 ± 146 and 175 ± 147 nS, average macroscopic junctional conductances. Ventricular myocyte pairs showed a single population of 100 pS, and atrial myocyte pairs, two populations of 100 and 185 pS single gap junction channel conductances (Verheule et al., 1997). Membrane potential change in an active cell thereby electrotonically depolarises the adjacent, initially quiescent, cell within the syncytium, with implications for conduction velocity in turn predisposing to pro-arrhythmic re-entrant circuits. Thus, in both experimental and clinical pro-arrhythmic pathological situations, remodelling gap junction relocation to lateral as opposed to perinexal membrane (Peters et al., 1997; Vetterlein et al., 2006) occurs with the acute ischaemia following coronary occlusion (Severs et al., 2008), advanced chronic ischaemic cardiac disease (Smith et al., 1991), cardiac failure arising from ischaemic, dilated and inflammatory cardiomyopathy (Kitamura et al., 2002; Kostin et al., 2003; 2004), and systemic or pulmonary hypertensive conditions (Kostin et al., 2004). Similarly, albeit likely multifactorial in pathophysiology, atrial fibrillation is associated with altered Cx40 expression and/or its lateral distribution (Polontchouk et al., 2001; Gemel et al., 2014; Lambiase and Tinker, 2015). Finally, though viable, adult Cx40-deficient, Cx40^−/−^/Cx43^+/+^ and Cx43-heterozygous, Cx40^+/+^/Cx43^+/−^mouse hearts show conduction abnormalities. Double heterozygous Cx40^+/−^/Cx43^+/−^showed ECG features suggesting additive Cx40 and Cx43 haploinsufficiency effects on ventricular, but not atrial, conduction (Kirchhoff et al., 2000).

However, propagation of myocardial excitation can persist under circumstances of reduced gap junction coupling. This is exemplified by clinical pathological conditions following ischaemic insult (Rohr, 2004), atrial fibrillation (Chaldoupi et al., 2009) and experimental platforms with loss-of-function, Cx43 (Eloff et al., 2001; Gutstein et al., 2001) or Cx40, genetic modifications (Hagendorff et al., 1999; Bagwe et al., 2005). These findings prompted suggestions of additional or alternative excitation propagation mechanisms including direct cell-to-cell, ephaptic, conduction (Sperelakis and Mann, 1977; Carmeliet, 2019). Widespread such examples occur in peripheral and central nervous systems of numerous species. They can mediate cell-cell, bidirectional or unidirectional excitation (Furshpan and Potter, 1959), either alone (Watanabe and Grundfest, 1961; Bennett, 1977) or combined with other complementary mechanisms (Rash et al., 1996).

### 1.2 Perinexal regions between cardiomyocytes

**FIGURE 1:**
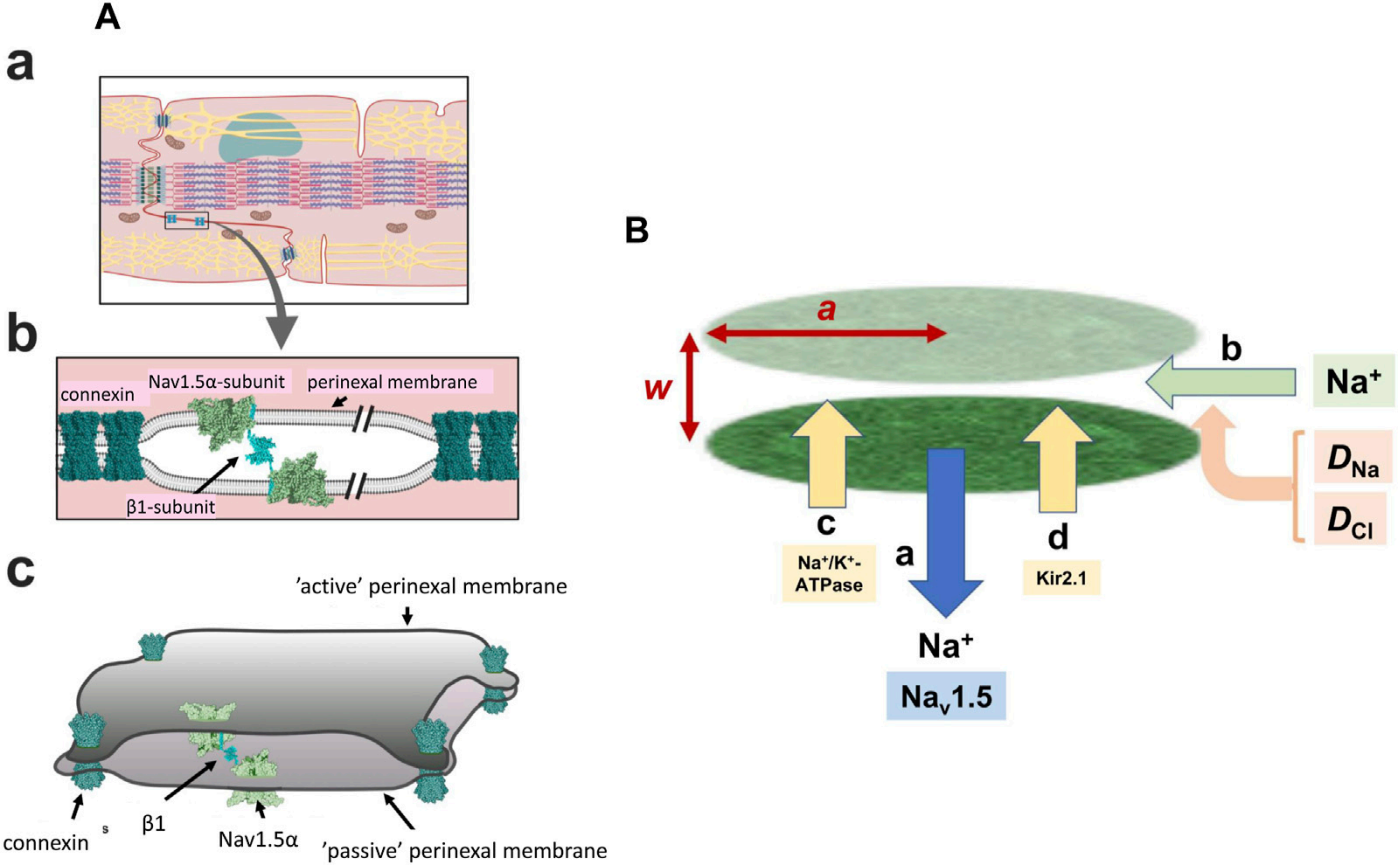
*Background geometrical parameters describing the model*. **(A)** The intercalated disc **(a)** and ephaptic junction **(b)** with three-dimensional arrangement of the intercalated disc **(c)** gap-junctions hold the membranes of two adjacent cells in close apposition with Na_v_ 1.5 β-subunits restricting the axial distance in other regions Reproduced from Salvage et al. (2020a), licensed under CC-BY 4.0. **(B)** Simplified physical scheme describing recovery processes at the ephaptic junction axial distance, *w*, radius, *a* containing Na^+^ and Cl^−^ with diffusion coefficients *D*_Na_, *D*_Cl_, following initial Na^+^ withdrawal consequent upon action potential generation **(a)**. This lists electrodiffusive **(b)** and other, Na^+^-K^+^-ATPase **(c)** and Kir2.1 **(d)** mediated membrane transport contributions to ionic composition and consequent membrane potential in the ephaptic space.

In the heart, adjacent cardiomyocytes are joined end-to-end at specialised perinexal regions (Figure 1Aa). These contain structural specialisations compatible with ephaptic function (Rhett and Gourdie, 2012; Rhett et al., 2013; Veeraraghavan et al., 2014). Electron microscopic appearances indicated closely apposed adjacent cell membranes (Veeraraghavan et al., 2015). These showed elevated Cx43 and Na^+^ channel (Nav1.5) expression. Super-resolution microscopy localised high Nav1.5 densities within 200 nm of the GJ plaques (Veeraraghavan et al., 2015; Hichri et al., 2018; Struckman et al., 2021), likely reflecting recently demonstrated intrinsic Nav1.5 clustering properties (Salvage et al., 2020b), also reported for skeletal muscle Nav1.4 (Sheikh et al., 2001). Smart patch clamping correspondingly indicated greater Na^+^ current densities (*I*_Na_) at perinexal than at non-junctional sites (Veeraraghavan et al., 2018). Such high, intercalated disk, Nav1.5 densities might thus support ephaptic conduction.

Super-resolution imaging additionally demonstrated preferential perinexal β1 (SCN1B) subunit colocation with the Nav1.5 (Veeraraghavan et al., 2018). These could form adhesion *trans* scaffolds adjacent to the gap junctions across the consequently narrow (∼20 nm) perinexal clefts (Salvage et al., 2020a) (Figure 1Ab). βadp1 peptide selectively inhibited this β1-mediated adhesion and widened guineapig ventricular perinexi. It selectively reduced perinexal but not whole cell *I*_Na_ precipitating arrhythmogenic conduction slowing (Veeraraghavan et al., 2018). These findings together suggested a Nav1.5 mediated ephaptic cell-cell transfer of electrical excitation, potentially offering a novel therapeutic anti-arrhythmic target. This could explain persistent myocardial electrical conduction in mouse models where electrical coupling was decreased by Cx43 knockout (Gutstein et al., 2001).

### 1.3 Previous modelling of ephaptic activation

Mathematical modelling suggested that ephaptic mechanisms could mediate AP conduction despite compromised gap junctional coupling (Sperelakis and Mann, 1977; Sperelakis, 2002; Mori et al., 2008). Ephaptic activation then provided a slower conduction requiring the nevertheless experimentally reported narrow perinexal axial distance (cleft width) and high Nav1.5 densities. The perinexal space constitutes a restricted extracellular diffusional space permitting formation of microdomains containing reduced [Na^+^] (Veeraraghavan et al., 2015). These could result from Na^+^ withdrawal during the AP upstroke of the initially active cell. The consequent negative potential within the resulting Gaussian space could depolarise the membrane of the adjacent, initially quiescent, cell. Should the latter then attain its Nav1.5 activation threshold it initiates an AP (Sperelakis, 2002). Additionally, gated stimulated emission depletion (gSTED) and stochastic optical reconstruction microscopy (STORM) super-resolution microscopy, attribute a significant perinexal K^+^ conductance to Kir2.1 expression particularly associated with desmosomes (Veeraraghavan et al., 2016; Struckman et al., 2021). Its marked inward rectification property reducing K^+^ conductance on depolarisation (Difranco et al., 2015; Chen et al., 2018) would enhance ephaptic excitation by minimising electrical shunting of the Na^+^ transfer. Existence of ephaptic activation between adjacent cardiomyocytes involving capacitive coupling of the closely apposed membranes thus has both experimental and theoretical support (Mori et al., 2008; Lin and Keener, 2010).

### 1.4 Requirements for ephaptic recovery

However, sustained operation of the resulting ephaptome also requires complete ***recovery*** of the altered extracellular ephaptic ionic concentration within each cardiac cycle. On the one hand, this could involve actions of other perinexal membrane molecules. As well as the increased Nav1.5 densities in intercalated disk regions, and Kir2.1 particularly around desmosomes, recent confocal microscopy and super resolution studies reported increased signals reflecting Na^+^-K^+^-ATPase occurring around the gap and adherence junctions (Figure 1B) (Noël et al., 1991; Mcdonough et al., 1996; Struckman et al., 2021). These patterns were more marked in atrial relative to ventricular myocytes. Finally, electrodiffusive exchanges particularly involving the principal extracellular ions Na^+^ and Cl^−^ could occur within the ephaptic space, and between the ephaptic space and the remaining extracellular space. Available information bearing on 3-dimensional perinexal geometry (Rhett and Gourdie, 2012; Gourdie, 2019) suggests that gap junction plaques forming relatively large regions of 100s of gap junctions surrounded by perinexal regions containing Nav1.5 clusters (Salvage *et al.*, 2020*b*) ‘pin’ together the component membranes at discrete sites (Figure 1Ac) (Salvage *et al.*, 2020*a*). However, they leave an ephaptic space offering pathways for diffusion around them. This permits diffusive interconnections between different ephaptic regions within the intercalated disc, as well as to the bulk extracellular fluid, possibly exerting some restriction to free diffusion. These could contribute to dissipation of the resulting ephaptic membrane potential change. Thus, acute interstitial oedema (AIE)-induced perinexal swelling produced an arrhythmogenic conduction slowing respectively mitigated and exacerbated by Kir2.1 inhibition and Nav1.5 block (Veeraraghavan et al., 2016). In addition, such recovery times have implications for the effectiveness of possible ephaptic conduction contributions at different heart rates (see Discussion).

### 1.5 Modelling electrodiffusive recovery

The present computational studies assess limiting features of such electrodiffusive ***recovery*** processes following AP activation and their variation with both ephaptic junction geometry and diffusional properties, for the first time. The modelling (1) employed experimentally reported values describing ephaptic junction anatomy in mammalian atria, ventricles and purkinje fibres and established initial values of extracellular electrolyte, Na^+^, Cl^−^, concentrations and their diffusion coefficients (Schoenberg et al., 1975; Severs, 2000; Mori et al., 2008; Richards et al., 2011; Veeraraghavan et al., 2015; 2018). It then (2) reproduced changes in these within the restricted ephaptic space resulting from a Δ*V*∼130 mV AP upstroke at physiological (37°C) temperatures and (3) followed their subsequent recovery. The latter involved determination of ionic concentrations and their variation with time and through the three dimensional ephaptic geometry (Léonetti, 1998; Koch, 2004) employing a finite element Nernst-Planck modelling of the consequent extracellular Na^+^ and Cl^−^ electrodiffusional processes. (4) The consequent membrane potential changes resulting from the imbalance in ion charges with finite diffusion coefficients within a restricted extracellular space were derived by an adaptation of Gauss’s Flux Theorem. Such a charge difference approach was introduced in previous membrane potential modelling studies from properties of multiple channel components (Fraser and Huang, 2004; 2007). This made it possible (5) to explore the robustness of the solutions and the effect of biological variations in ephaptic dimensions, membrane anatomy, and diffusion restrictions. These features made it possible to consider potential comparative roles of electrodiffusive and membrane transport processes in ephaptic recovery in different, atrial, ventricular and purkinje cardiomyocyte types in relationship to known physiological heart rates in different species.

## 2 Theory

### 2.1 Forms of the Nernst-Planck equation describing three-dimensional electrodiffusional fluxes

Solving for the time and spatial dependence of ephaptic ion concentrations, *c*, electrodiffusionally recovering following initial Na^+^ withdrawal employed the fundamental time (*t*) dependent Nernst-Planck equations. This relates flux, *J*, to concentration *c*, and electrical potential φ for an ion with diffusion coefficient, *D* and valence, *z* assuming Faraday constant, *F*, gas constant, *R*, and absolute temperature, *T*:

$$J=-D\left[\nabla c+\frac{zF}{RT}c\left(\nabla \varphi \right)\right].$$ (1)

Substituting [1] in the conservation relationship:

$$\frac{\partial c}{\partial t}=-\nabla .J$$ (2)

Gives:

$$\frac{\partial c}{\partial t}=\nabla .D\left[\nabla c+\frac{zF}{RT}c\left(\nabla \varphi \right)\right]$$ (3)

Applying the product rule and collecting terms to the left hand side of the equation:

$$\frac{\partial c}{\partial t}-\nabla .\left(D\nabla c\right)-\;D\frac{zF}{RT}\left(c\right.\nabla^{2}\varphi )-D\frac{zF}{RT}\left(\nabla c.\;\nabla \varphi \right)=0$$ (4)

Splitting the Nernst Planck Equation into two equations representing positive (+ve) and negative (-ve) ions:

$$\frac{\partial c^{+ve}}{\partial t}-D^{+ve}\nabla^{2}c^{+ve}-D^{+ve}\frac{zF}{RT}c^{+ve}\nabla^{2}\varphi -\;D^{+ve}\frac{zF}{RT}\nabla \varphi .\;\nabla c^{+ve}=0$$ (5)

$$\frac{\partial c^{-ve}}{\partial t}-D^{-ve}\nabla^{2}c^{-ve}-D^{-ve}\frac{zF}{RT}c^{-ve}\nabla^{2}\varphi -\;D^{-ve}\frac{zF}{RT}\nabla \varphi .\;\nabla c^{-ve}=0$$ (6)

### 2.2 Initial action potential mediated Na^+^ transfer from the ephaptic space

These equations were solved for an ephaptic space comprising a circularly symmetric gap, radius *a* and axial separation *w,* filled with extracellular fluid, free-space permittivity ε_0,_ and water dielectric constant ε_c_, containing principal charge carriers Na^+^ and Cl^−^. Their initial conditions modelled effects of an AP upstroke withdrawing [Na^+^] from this space uniformly through the *active* membrane, perturbing ionic concentration differences within the ephaptic space. Derivation of limiting minimum values for this Na^+^ transfer employed active membrane surface area:

$$S_{AP}=\pi a^{2}.$$ (7)

Assuming specific membrane capacitance, *C*_m_, the Na^+^ current then discharges capacitance,

$$\pi a^{2}C_{m}.$$ (8)

The charge *q* transferred across the active membrane, by membrane potential change Δ*V* is then:

$$q=\left(\Delta V\right)\pi a^{2}C_{m}$$ (9)

The charge of a single, univalent (*z* = +1 or −1) Na^+^ or Cl^−^ ion equals the elementary charge *e =*1.6021 × 10^−19^ C. Assuming Avogadro’s number *N*_A_ = 6.0221367 × 10^23^ mol^-1^ (Lide, 1993), the molar Na^+^ transferred from the ephaptic space is therefore:

$$Moles\;of\;{Na}^{+}\;transferred=\frac{\left(\Delta V\right)C_{m}\pi a^{2}}{eN_{A}}$$ (10)

For example, for atrial cells with typical ephaptic radius *a* = 8 × 10^−6^ m and axial distance *w* = 20 × 10^−9^ m, and specific plasma membrane capacitance *C*_m_ = 0.01 F m^-2^, a membrane potential change Δ*V* = +130 × 10^−3^ V (Table 1, see also (Draper and Weidmann, 1951; Hilgemann and Noble, 1987)**)**, then transfers 2.7090 × 10^−18^ mol Na^+^ from an initially neutral ephaptic space.

**TABLE 1:** Basic parameters for cardiac ephaptic junctions.

Parameter	Value	Reference
Plasma membrane specific capacitance, *C*_m_ (μF cm^-2^)	1.0	Satoh et al. (1996)
Action potential upstroke voltage (V)	1.30 × 10^−1^	Draper and Weidmann (1951), Hilgemann and Noble (1987)
Atrial ephaptic radius, *a* (nm)	8 × 10^3^	Richards et al. (2011)
Ventricular ephaptic radius, *a* (nm)	12 × 10^3^	Severs, 2000; Mori et al. (2008)
Purkinje ephaptic radius, *a* (nm)	40 × 10^3^	Schoenberg et al. (1975)
Axial distance, *w* (nm)	20	Veeraraghavan et al. (2015)
Na^+^ diffusion coefficient, *D*_Na_ (nm^2^ s^-1^)	1.3 × 10^9^	Passiniemi (1983)
Cl^−^ diffusion coefficient, *D*_Cl_ (nm^2^ s^-1^)	2 × 10^9^	Passiniemi (1983)
Dielectric constant of water at 37°C (ε_c_)	74.3	Malmberg and Maryott (1956), Gabriel et al. (1996), Kramlich et al. (2012)
Free-space permittivity, ε_0_ (F nm^-1^)	8.85418 × 10^−21^	Lide (1993)
Elementary charge, *e* (C)	1.60218 × 10^−19^	Lide (1993)
Avogadro’s constant, *N*_A_ (mol^-1^)	6.02214 × 10^23^	Lide (1993)
Faraday constant, *F* (C mol^-1^)	9.64853 × 10^4^	Lide (1993)
Molar gas constant, *R* (J mol^-1^ K^−1^)	8.31451	Lide (1993)
Temperature, *T* (K)	310.15	Hutchison et al. (2008)
Resting Na^+^ concentration, [Na^+^] (mM)	153.1	Trautwein et al. (1962), Dijkman et al. (1997), Lai et al. (2007)
Resting Cl^−^ concentration [Cl^−^] (mM)	145.8	Trautwein et al. (1962), Dijkman et al. (1997), Lai et al. (2007)

### 2.3 Gaussian determination of the voltage recovery from changes in the ion concentration difference

The Nernst-Planck electrodiffusion analysis then examined spatial and temporal changes in ephaptic space ionic concentrations and their consequences for membrane potential following the initial ephaptic excitation. The ephaptic space was approximated to two closely apposed, active and passive, membranes radius *a* separated by small ephaptic gap distance *w* whose margins access the bulk extracellular compartment (Figure 1B),

w≪a. (11)

The membrane areas (Eq. 12) therefore greatly exceed that of the rim of the ephaptic gap:

$$2\pi aw\ll \pi a^{2}.$$ (12)

Following action potential recovery and early rapid axial diffusive movements these might first produce an axially and radially uniform ionic concentration change, and radially uniform membrane potential and electric field changes across the enclosing plasma membranes. The subsequent electrodiffusive recovery primarily involving fluxes of the principal extracellular ions Na^+^ and Cl^−^ then result in the time and position dependent [Na^+^] and [Cl^−^] of *c* ^+ve^ and *c* ^-ve^ respectively. The consequent time and spatially dependent radial ionic concentration differences {*c* ^+ve^ - *c* ^-ve^} within the ephaptic space cause correspondingly time and spatially dependent membrane potential alterations, ∆*V*. Gauss’s Flux Theorem gives the electric flux ${\delta \Phi }_{\hat{Ε}}$ given by the surface integral of the electric field *Ê* over any closed area *S* generated by enclosed net charge δ *q* in medium of relative permittivity $\varepsilon_{r}$ (Lide, 1993):

$$\delta \Phi_{\hat{Ε}}\;=∯\;\delta \hat{Ε}.\;dS\;=\frac{\delta q}{\varepsilon_{0}\varepsilon_{r}}$$ (13)

Consider a surface falling within, and in which most of whose electrical flux traverses, the plasma membranes of relative permittivity $\varepsilon_{r},$ enclosing the ephaptic space. As 2π*aw* << π*a* ^2^ (Equation (12)), and $\varepsilon_{r}$ << $\varepsilon_{c}$ , the position and time-dependent transmembrane membrane potential change ∆*V* tends to the corresponding φ within the adjoining recovering ephaptic space. The latter geometrically approximates a series of successive coaxial, circularly symmetric, concentric annular volume elements. Each element contains progressively altered {*c* ^+ve^ - *c* ^-ve^}. These charge membranes of permittivity ε forming the two axial ends of each element. The total membrane cross-sectional *area* δS in any given annular element of inner radius α and outer radius $\left(\alpha \right.$ + δ α ), is:

$$\delta S=2\left\{\pi {\left(\alpha +\delta \alpha \right)}^{2}-\pi \alpha^{2}\right\}\;=2\pi \left(2\alpha +\delta \alpha \right)\left(\delta \alpha \right)\;=4\pi \alpha \delta \alpha +2\pi \delta \alpha^{2}$$ (14)

The *volume* of each annular element in a ephaptic gap of axial width (*w*) is then:

$$\frac{w\delta S}{2}$$ (15)

The *concentration* of charge, [*q*] within that volume element is the product of the ionic concentration difference, $\left\{c^{+ve}-c^{-ve}\right\}$ , and Faraday’s constant (*F*), for Na^+^ and Cl^−^ ions of valencies *z* = +1 and −1 respectively:

$$\left[q\right]\;=zF\left\{c^{+ve}-c^{-ve}\right\}$$ (16)

The corresponding *quantity* of charge present within that element, δ *q*, is then the concentration of the charge $\left[q\right]$ multiplied by its volume. From equations (15) and (16):

$$\delta q=\frac{wzF\delta S}{2}\left\{c^{+ve}-c^{-ve}\right\}$$ (17)

In the limit δα→0, adjoining annular elements tend towards equal δS , each containing equal charge δ *q* minimizing the proportion of the total electric flux between elements. Most of the flux then traverses the ephaptic membranes at the two ends of each element. For membranes of thickness, *ζ*, total surface area *S*, whose electric field is uniform (Goldman, 1943), Δ *Ê =* φ */ζ*:

$${\Delta \Phi }_{Ê}=∯\Delta Ê.dS=\frac{\varphi }{\zeta }\delta S$$ (18)

Equating the right sides of Eqs. (13), (18) gives the alteration in transmembrane voltage:

$$\varphi =\frac{\zeta \delta q}{\varepsilon_{0}\varepsilon_{r}\delta S}=\frac{\delta q}{C_{m\;}\delta S},$$ (19)

since:

$$C_{m\;}=\frac{\varepsilon_{0}\varepsilon_{r}}{\zeta }$$ (20)

Finally, substituting the expressions (Eq. 17) for δ *q*:

$$\varphi =\frac{zFw\left\{c^{+ve}-c^{-ve}\right\}}{{2C}_{m}}$$ (21)

This gives:

$$\nabla \varphi =\nabla \left(\frac{zFw\left\{c^{+ve}-c^{-ve}\right\}}{{2C}_{m}}\right)=\frac{zFw}{{2C}_{m}}\nabla \left(\left\{c^{+ve}-c^{-ve}\right\}\right)$$ (22)

This treatment employing Gauss’s Flux theorem extends previous analyses deriving cell resting potentials from intracellular charge concentration differences (Fraser and Huang, 2004; 2007). The latter studies applied Eq. (19) to a cell of volume ϑ containing *homogeneous* intracellular electrolyte and protein concentrations ${\left[{\text{Na}}^{+}\right]}_{i},{\left[K^{+}\right]}_{i},{\left[{\text{Cl}}^{-}\right]}_{i}\;\text{and }{\left[{\mathrm{Pr}}^{z-}\right]}_{i}$ and consequently *uniform* resting membrane potential φ . The $\frac{\delta q}{\delta S}$ term becomes the ratio between total charge difference related to the intracellular (*i*) ion concentrations, $\vartheta F\left({\left[{\text{Na}}^{+}\right]}_{i}+{\left[K^{+}\right]}_{i}-{\left[{\text{Cl}}^{-}\right]}_{i}+z_{x}{\left[{\mathrm{Pr}}^{z-}\right]}_{i}\right),$ and total membrane surface area *S* (Fraser and Huang, 2004). The ratio between the resulting total surface membrane capacitance *SC*_m_ and the ϑ term gives the capacitance of unit cell volume $C_{\vartheta }$ yielding a resting potential equation previously derived by alternative means (Fraser and Huang, 2004):

$$\varphi =\frac{F\left({\left[{\text{Na}}^{+}\right]}_{i}+{\left[K^{+}\right]}_{i}-{\left[{\text{Cl}}^{-}\right]}_{i}+z_{x}{\left[{\mathrm{Pr}}^{z-}\right]}_{i}\right)}{C_{\vartheta }}$$ (23)

### 2.4 Computational implementation of the Nernst-Planck formalisms

Combining Eqs. (5), (6), (22) and the Poisson Equation (Malmberg and Maryott, 1956; Gabriel et al., 1996; Kramlich et al., 2012):

$$\nabla^{2}\varphi =\frac{e}{\varepsilon_{0}\varepsilon_{c}}\left[c^{-ve}-c^{+ve}\right]$$ (24)

yielded analytic equations for the present specific Nernst-Planck diffusion process over time for numerical solution by finite element analysis (FEA). For the univalent Na^+^ (*z* = 1) and Cl^−^ (*z* = −1):

For $c^{+ve}$ (*z* = +1):

$$\frac{\partial c^{+ve}}{\partial t}-D^{+ve}\nabla^{2}c^{+ve}-D^{+ve}\frac{Fe}{RT\varepsilon_{0}\varepsilon_{c}}c^{+ve}\left[c^{-ve}-c^{+ve}\right]-D^{+ve}\frac{F^{2}w}{RT{2C}_{m}}\nabla \left(\left\{c^{+ve}-c^{-ve}\right\}\right).\nabla c^{+ve}=0$$ (25)

For $c^{-ve\;}$ (*z* = −1):

$$\frac{\partial c^{-ve}}{\partial t}-D^{-ve}\nabla^{2}c^{-ve}+D^{-ve}\frac{Fe}{RT\varepsilon_{0}\varepsilon_{c}}c^{-ve}\left[c^{-ve}-c^{+ve}\right]+D^{-ve}\frac{F^{2}w}{RT{2C}_{m}}\nabla \left(\left\{c^{+ve}-c^{-ve}\right\}\right).\nabla c^{-ve}=0$$ (26)

These can be re-arranged into a form solvable by the matrix-based MATLAB platform:

$$\lambda_{1}\frac{\partial^{2}c}{\partial t^{2}}+\lambda_{2}\frac{\partial c}{\partial t}-\nabla .\left(\lambda_{3}\nabla c\right)+\lambda_{4}c\;-\;\lambda_{5}\;=0$$ (27)

Thus, applying the product rule:

$$\nabla .\left(\lambda_{3}\nabla c\right)\;=\nabla \lambda_{3}.\nabla c\;+\;\lambda_{3}\nabla^{2}c$$ (28)

gives:

$$\lambda_{1}\frac{\partial^{2}c}{\partial t^{2}}+\lambda_{2}\frac{\partial c}{\partial t}-\lambda_{3}\nabla^{2}c-\nabla \lambda_{3}.\nabla c\;+\;\lambda_{4}c\;-\;\lambda_{5}\;=0$$ (29)

If λ_3_ is constant, $\nabla \lambda_{3}.\nabla c=0,$ as confirmed in Eqs. (31), (32) below:

$$\lambda_{1}\frac{\partial^{2}c}{\partial t^{2}}+\lambda_{2}\frac{\partial c}{\partial t}-\lambda_{3}\nabla^{2}c\;+\;\lambda_{4}c\;-\;\lambda_{5}\;=0$$ (30)

The partial differential equations (PDEs) were solved using the Partial Differential Equation (PDE) Toolbox™. The coefficients $\lambda_{1}$ , $\lambda_{2}$ …. $\lambda_{5}$ can be functions of location (*x*, *y*, and, in 3-D, *z*), and, except for eigenvalue problems, can also be functions of the solution *c* or its gradient. Thus, comparing terms between equation (25) and (30), for $c^{+ve}$ :

$$\begin{array}{c} \lambda_{1}\;=0\\ \lambda_{2}\;=1\\ \lambda_{3}=D^{+ve}\\ \lambda_{4}\;=-D^{+ve}\frac{Fe}{RT\varepsilon_{0}\varepsilon_{c}}\left[c^{-ve}-c^{+ve}\right]\\ \lambda_{5}=D^{+ve}\frac{F^{2}w}{RT{2C}_{m}}\nabla \left(\left\{c^{+ve}-c^{-ve}\right\}\right).\nabla c^{+ve} \end{array}$$ (31)

Comparing terms between Eqs. (26), (30), for $c^{-\text{ve}}$ :

$$\begin{array}{c} \lambda_{1}\;=0\\ \lambda_{2}\;=1\\ \lambda_{3}=D^{-ve}\\ \lambda_{4}\;=-D^{-ve}\frac{Fe}{RT\varepsilon_{0}\varepsilon_{c}}\left[c^{-ve}-c^{+ve}\right]\\ \lambda_{5}={-D}^{-ve}\frac{F^{2}w}{RT{2C}_{m}}\nabla \left(\left\{c^{+ve}-c^{-ve}\right\}\right).\nabla c^{-ve} \end{array}$$ (32)

## 3 Materials and methods

The Nernst-Planck diffusion of Na^+^ and Cl^−^, and their subsequent patterns of temporal and spatial ion concentration and membrane potential differences were examined in a computational model of cardiac ephaptic junctions. Their anatomy was replicated using published values reported for mammalian atrial, ventricular, and purkinje cardiomyocytes. Further computations were also applied following proportional changes in the axial distances and radii of the junctions examined, and diffusion coefficients defining the underlying flux processes. The analysis modified a previous approach examining diffusional fluxes in Ca^2+^ microdomain formation at skeletal muscle triad junctions (Bardsley et al., 2021). This used the matrix-based MATLAB language, implemented on the MATLAB platform, in a finite element analysis (FEA) of ion diffusion over time. The partial differential equations (PDEs) involved were solved using the Partial Differential Equation (PDE) Toolbox™, modifying the relevant code to model Nernst-Planck diffusion.

The studies first involved meshing the geometry and selection of physical conditions (initial and boundary conditions). This permitted generation of a set of solution matrices for subsequent processing and figure presentation. The studies used the MATLAB matrix-based programming platform and language (version R2021b win64 9.11.0.1769968, version released 22 September 2021, MathWorks, Cambridge, UK) (https://www.mathworks.com/discovery/what-is-matlab.html). The programmes performed the required data array manipulations and generated all the graphics shown in the results section excluding Figure 8 which was produced using SciDAVis (version 2.4, version released fifth May 2021, developed by Miquel Garriga, Arun Narayanankutty, Dmitriy Pozitron, Russel Standish). This modelling was performed using an IBM compatible computer (CPU: Intel® Core™ i5-8265U CPU@1.60GHz; installed RAM: 8 GB, running Windows (Microsoft, Washington, USA) 11, Home 64-bit version 21H2).

The ephaptic junction geometry was generated within MATLAB and then divided into a set of tetrahedral sub-domains (elements) joined along their edges and at vertices (nodes), to be used in a FEA solving a set of PDEs. This culminated with a representation of the global solution whilst retaining local effects. We made use of both Neumann-Type and Dirichlet-Type boundary conditions for the modelling of ephaptic junction recovery which provide values of the field variables at the boundaries of our geometry. FEA equations are used to calculate the value of the field variable at nodes, interpolation of these values is then used to calculate values for non-nodal points (Rapp, 2017). Where two tetrahedra connect (vertices and edges) the value of the field variable is the same between the two elements. This ensures continuity between them, preventing gaps in the solution. Each element represents our set of PDEs, which are time-dependent, with a set of ordinary differential equations (ODEs) that approximate the solution to the original equations (Ramirez, 1997). This set of ODEs is then numerically integrated by MATLAB solvers to provide a solution, for which a source listing is provided in the Supplementary file.

Meshing and the FEA performed used the PDE Toolbox™ (version 3.7 installed on 27 September 2021 by MathWorks) within MATLAB. As indicated above, the PDE Toolbox™ requires PDEs to be in the form:

$$\lambda_{1}\frac{\partial^{2}u}{\partial t^{2}}+\lambda_{2}\frac{\partial u}{\partial t}-\nabla .\left(\lambda_{3}\nabla u\right)+\lambda_{4}u=\lambda_{5}$$ (27)

Where the solution *u* is the concentration of a given ion, and $\lambda_{1}$ , $\lambda_{2}$ …. $\lambda_{5}$ are inputted coefficients. This automatically meshes the ephaptic junction geometry given a maximum mesh size (maximum edge length), providing a platform for implementing our set of PDEs. It then outputs and stores the solutions as matrices. These could then be accessed for processing of the raw-data and subsequent presentation in MATLAB.

## 4 Results

### 4.1 Na^+^ transfer across the active membrane: Initial conditions for computational modelling

The finite element analysis (FEA) employing the PDE Toolbox™ modelled electrodiffusive ion fluxes through stepsizes representing both its spatial and temporal finite elements. The ephaptic geometry was meshed into tetrahedral elements joined along their edges and at their vertices (nodes) where the side length, defining the mesh size, determined the spatial resolution of the computational solutions. In turn, the time step adopted for the solver gives the stepsize interval between successive iterations, therefore determining the temporal resolution. In general, smaller stepsize generate more accurate and stable solutions whilst requiring greater computer resources. Computational solutions were also obtained using combinations of varied spatial mesh and temporal stepsize to ensure appropriate optimisations between mathematical conditions.

Table 1 summarises the adopted, previously reported, physical and geometrical properties of the modelled atrial, ventricular and purkinje fibre ephaptic spaces and relevant physical constants. Values of atrial, ventricular and purkinje cardiomyocyte fibre radius, *a*, were obtained from previous experimental reports on hearts from large mammals (sheep). The selected values additionally permitted parallel investigations of the effect of variations in *a* on the computed solutions (Schoenberg et al., 1975; Severs, 2000; Richards et al., 2011) and comparisons with previous modelling results on ephaptic activation. Thus the value for ventricular radius was close to that used in recent reports (Mori et al., 2008). Values of axial ephaptic distance *w* employed recent, 15–30 nm, cell-cell distance measurements (Veeraraghavan et al., 2015; 2018). In any case, the investigations explored the effects of twofold alterations in *w* on the solutions. Similarly, details of membrane geometry that could affect Na^+^ and Cl^−^ diffusion were reflected in calculations varying their respective diffusion coefficients *D*_Na_ and *D*_Cl_. Thus, the wide range of *a*, *w, D*_Na_ and *D*_Cl_ investigated permitted a broad clarification of the effects of these parameters on the electrodiffusive recovery process. These parameters defined the geometrical boundaries of the space under analysis, specifically: the active membrane performing the initiating AP, the passive membrane responding to the resulting ephaptic conduction and the rim through which the ephaptic space, in which the consequent electrodiffusive processes under analysis take place, communicates with the surrounding extracellular space.

The computational solutions involved two stages. First, they were initiated by modelling an AP mediated Na^+^ transfer adopting a limiting minimum value for the Na^+^ transfer from the ephaptic space of radius *a* and axial width *w*. The magnitude of this Na^+^ transfer was determined by the Na^+^ flux produced by activation of evenly distributed, voltage-gated Nav1.5 channels responsible for driving the AP upstroke in the ***active*** membrane. This formal analysis assumed a membrane potential change *ΔV =* +130 mV (Draper and Weidmann, 1951; Hilgemann and Noble, 1987) across a plasma membrane, without infoldings, of specific capacitance *C*_m_ = 1 μF/cm^2^. Equations (7)–(10) permitted calculations of the charge *q* and moles of Na^+^ transferred across the active membrane of the ephaptic space. The biophysically derived value of Na^+^ flux was converted into a flux density referred to unit area of active ephaptic membrane surface. This provided the first, spatial, Neumann, boundary condition of the active ephaptic membrane. Boundary conditions at the ephaptic rim and the passive membrane were here set to zero indicating a zero ionic flux. The computational runs then determined the resulting axial and radial gradients of [Na^+^] and [Cl^−^] following this initial Na^+^ transfer.

**FIGURE 2:**
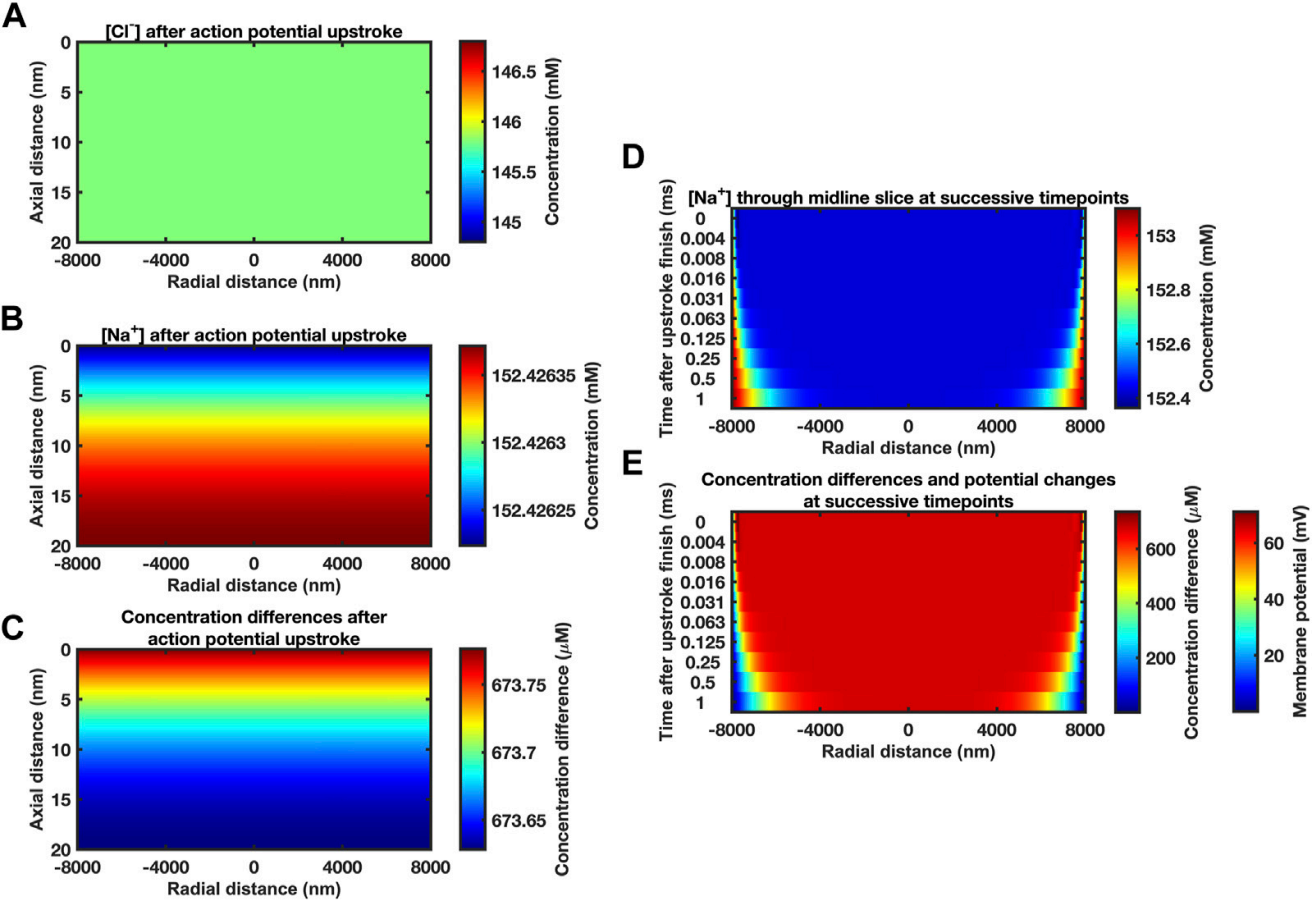
*Midline slice heat maps of concentration and voltage profiles close to the end of the action potential upstroke.* **(A–C)** Heat maps of [Cl^−^] **(A)** and [Na^+^] **(B)** and resulting ion concentration and membrane potential differences **(C)** at different axial (vertical axis) and radial positions (horizontal axis) in the atrial ephaptic space. Slices obtained at end of a 0.7 ms period of sodium extrusion representing the cardiac action potential upstroke. Note the very small axial gradients generated. Given the size of these gradients and the time-period of recovery to allow dissipation of these gradients we have assumed a uniform concentration profile for the initial conditions. Computational mesh size 400 nm. Stepsize interval 0.01 ms. **(D, E)** Series of midline slice heat maps at successive time points of recovery (left axis) over the first millisecond of recovery beginning from the initial conditions derived from the Na^+^ transfer established in **(A–C)**. **(D)** [Na^+^] and **(E)** ionic concentration differences. Scale bars on right: concentration differences **(D, E)** and membrane potential changes **(E)** at the passive membrane. Mesh size 400 nm; stepsize interval 0.001 ms.

Secondly, following the above 0.7 ms duration step, 2.7090 × 10^−18^ mol, Na^+^ transfer from the ephaptic space representing the AP upstroke, a Nernst-Planck electrodiffusion analysis examined limiting properties of ephaptic space [Na^+^] and [Cl^−^] recovery, and consequent membrane potential changes in the ***passive*** membrane. The Neumann boundary conditions of the active and passive membranes were now both set to zero representing zero flux during this recovery. The ephaptic rim was assigned Dirichlet boundary conditions holding [Na^+^] and [Cl^−^] at 153.1 mM and 154.8 mM respectively representing a well stirred bulk extracellular fluid of constant concentrations. Figure 2 illustrates early changes in atrial cells, ephaptic radius *a* = 8 × 10^−6^ m (Richards et al., 2011), axial distance *w* = 20 × 10^−9^ m (Veeraraghavan et al., 2018), and specific membrane capacitance *C*_m_ = 0.01 F m^-2^ and a Δ*V* = +130 × 10^−3^ V membrane potential change (Table 1). The computation employed a 400 nm mesh size and 0.01 ms stepsize interval. The initial 400 nm mesh size corresponded to 5% of the ephaptic radius, and the 0.01 ms stepsize interval entailed 100 iterations per ms time modeled. Figures 2A–C provides heat maps of axial (vertical axis) and radial (horizontal axis) [Cl^−^] (A) and [Na^+^] at mM (B) and the resulting charged ion concentration differences at higher μM, resolution (C) immediately following the Na^+^ transfer. Radial [Na^+^] gradients here were expectedly absent. Axial [Na^+^] and charged ion concentration differences, [*q*], were also extremely small and only detectable at high colour magnifications. The very small size of these gradients and the time-period of recovery to allow dissipation of these gradients permitted adoption of uniform axial concentration profiles in the initial conditions.

The latter conditions were confirmed in limiting properties of ephaptic space [Na^+^] and [Cl^−^] recovery and consequent passive membrane potential changes obtained over the first millisecond of the recovery with parameters in Table 1 at mesh size 400 nm and stepsize interval 0.001 ms. Figure 2D illustrates results of the Nernst-Planck electrodiffusion analysis for the limiting properties of the recovery of ephaptic space [Na^+^] and [Cl^−^]. Figure 2E portrays the consequent membrane potential changes in the ***passive*** membrane following the Na^+^ transfers produced by the initial ephaptic excitation. These confirm undetectable axial concentration gradients in contrast to time-evolving radial changes. Thus, additional to portraying changes within the first ms the following Na^+^ transfer, these findings suggest that the recovery process could be simulated assuming uniform concentration profiles for the initial conditions. Such additional simulations indeed yielded demonstrated concordant results (Supplementary Figure S1). This permitted a uniform concentration profile approximation in subsequent computations over longer time intervals to reduce computational time and resource requirements.

### 4.2 Electrodiffusive recovery in the atrial ephaptic space

**FIGURE 3:**
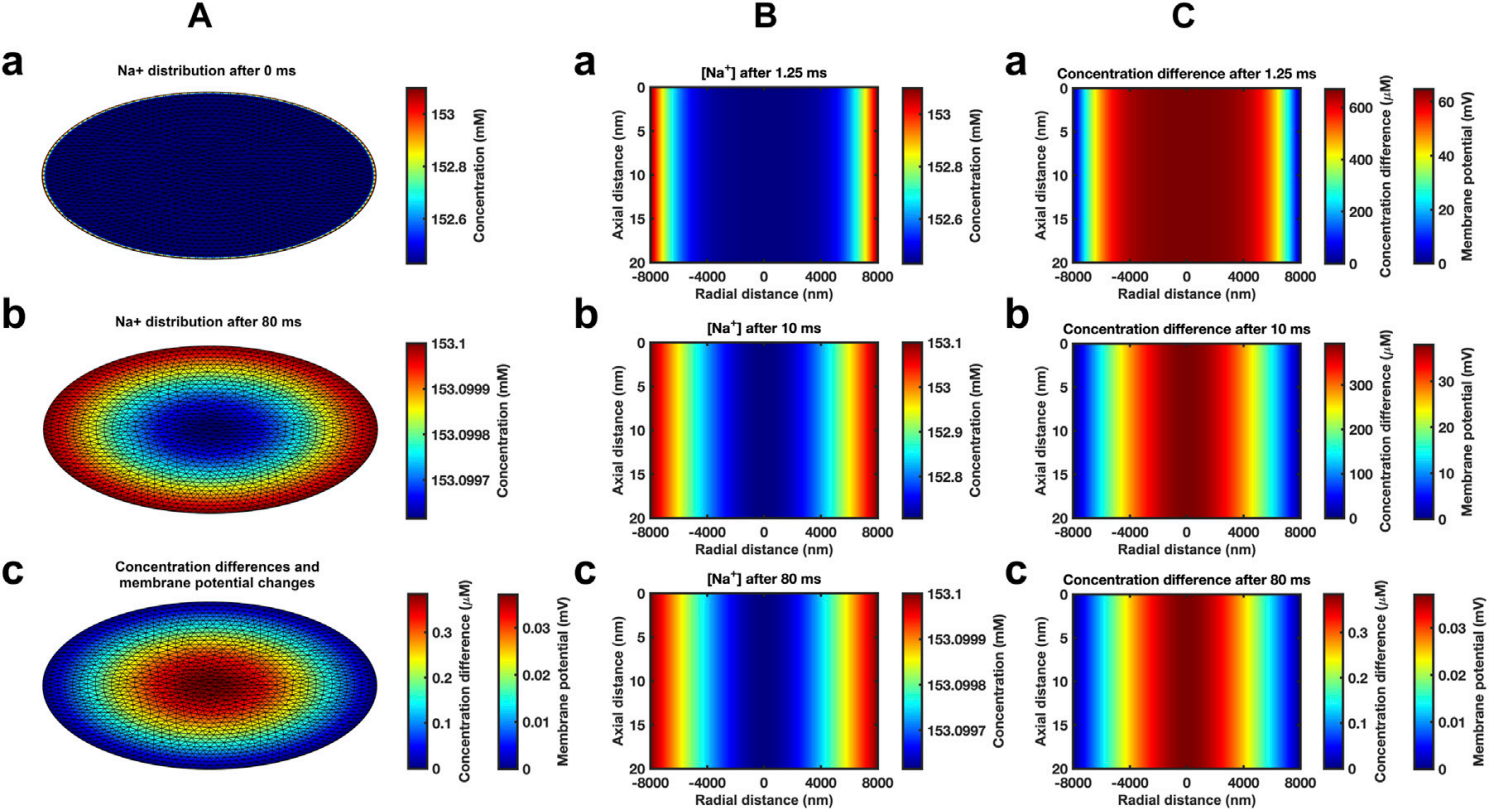
*Heat maps of atrial ephaptic recovery.* **(A)** Radial distributions of [Na^+^] **(a, b)** and of charged ion concentration differences **(c)** at the start **(a)** and end **(b, c)** of the recovery period. **(B, C)** Midline slice heat maps of [Na^+^] **(B)** and of charged ion concentration differences **(C)** at critical time points (1.25 ms, 10 ms, and 80 ms) in the atrial ephaptic space during recovery.

Modelling subsequent recovery timecourses accordingly adopted axially uniform initial ephaptic ion concentrations. Neumann boundary conditions of the active and passive membrane were now set to zero reflecting zero flux across these during recovery. Dirichlet boundary conditions set the remaining well stirred extracellular space [Na^+^] and [Cl^−^] at 153.1 mM and 154.8 mM respectively (Trautwein et al., 1962; Dijkman et al., 1997; Lai et al., 2007). The computation, exemplified in section D in the supplementary file, applied the atrial ephaptic parameters *a* = 8,000 nm, *w* = 20 nm (Table 1). It extended over a period covering 99.99975% recovery to resting [Na^+^] at the ephaptic centre in turn corresponding to a 99.94% ion concentration recovery. Cross-sectional (Figure 3A) heatmaps of [Na^+^] (Figures 3Aa,b) and ion concentration differences (Figure 3C) demonstrated uniform [Na^+^] through the radial plane at the outset (Fig. 3Aa). Maps shown following recovery at 80 ms (Figures 3Ab,c) demonstrated extremely small residual differences symmetrical along the radial direction. Midline sectional heatmaps (Figures 3B,C) of [Na^+^] (Figure 3B) and ion concentration differences (Figure 3C) at critical, 1.25 ms, 10 ms, and 80 ms, time-points during the recovery (a, b, c) demonstrated uniform values along the axial direction, and variations in the radial gradients during recovery.

**FIGURE 4:**
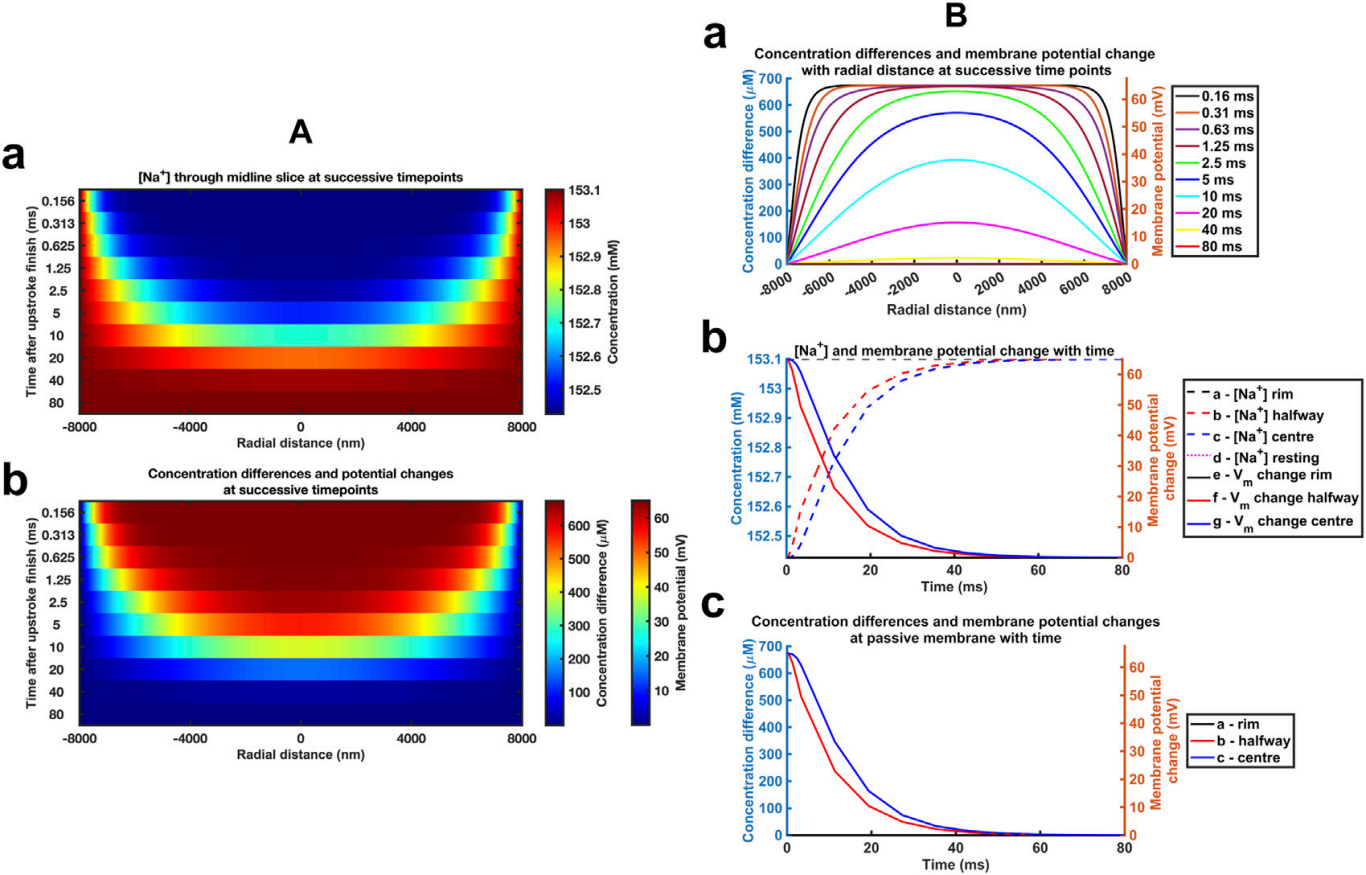
Time and spatial dependences of the atrial ephaptic recovery process*.* **(A)** Midline slice heat maps at successive time points of recovery representing **(a)** [Na^+^] and **(b)** ionic concentration differences and membrane potential changes. **(B)** Quantification of the temporal and spatial recovery. **(a)** [Na^+^] spatial profiles with time; Recovery timecourses of **(b)** [Na^+^] and membrane potential and **(c)** ionic concentration differences at the ephaptic rim, ephaptic centre and half-way between the two.

Graphical displays at successive logarithmically incremented time points during recovery provided more detailed representations of midline slice [Na^+^] (Figure 4Aa) and ionic charge concentration differences (Figure 4Ab), the corresponding membrane potential changes (Figure 4Ab) and their spatial profiles. The corresponding quantified graphs showed marked changes both in absolute values and spatial profiles of [Na^+^] and the corresponding membrane potential profiles with time (Figure 4Ba). The quantification also yielded detailed monotonic recovery timecourses of the [Na^+^] and membrane potential changes at the ephaptic rim, ephaptic centre and half-way between the two (Figure 4Bb). They also compared the ionic concentration differences with the membrane potential change (Figure 4Bc). Note that the Dirichlet boundary conditions would result in a constant ephaptic rim [Na^+^] = 153.1 mM and zero membrane potential changes. Within the ephaptic space the recovery followed an approximately exponential trend and was effectively complete within the 80 ms modelling interval.

**FIGURE 5:**
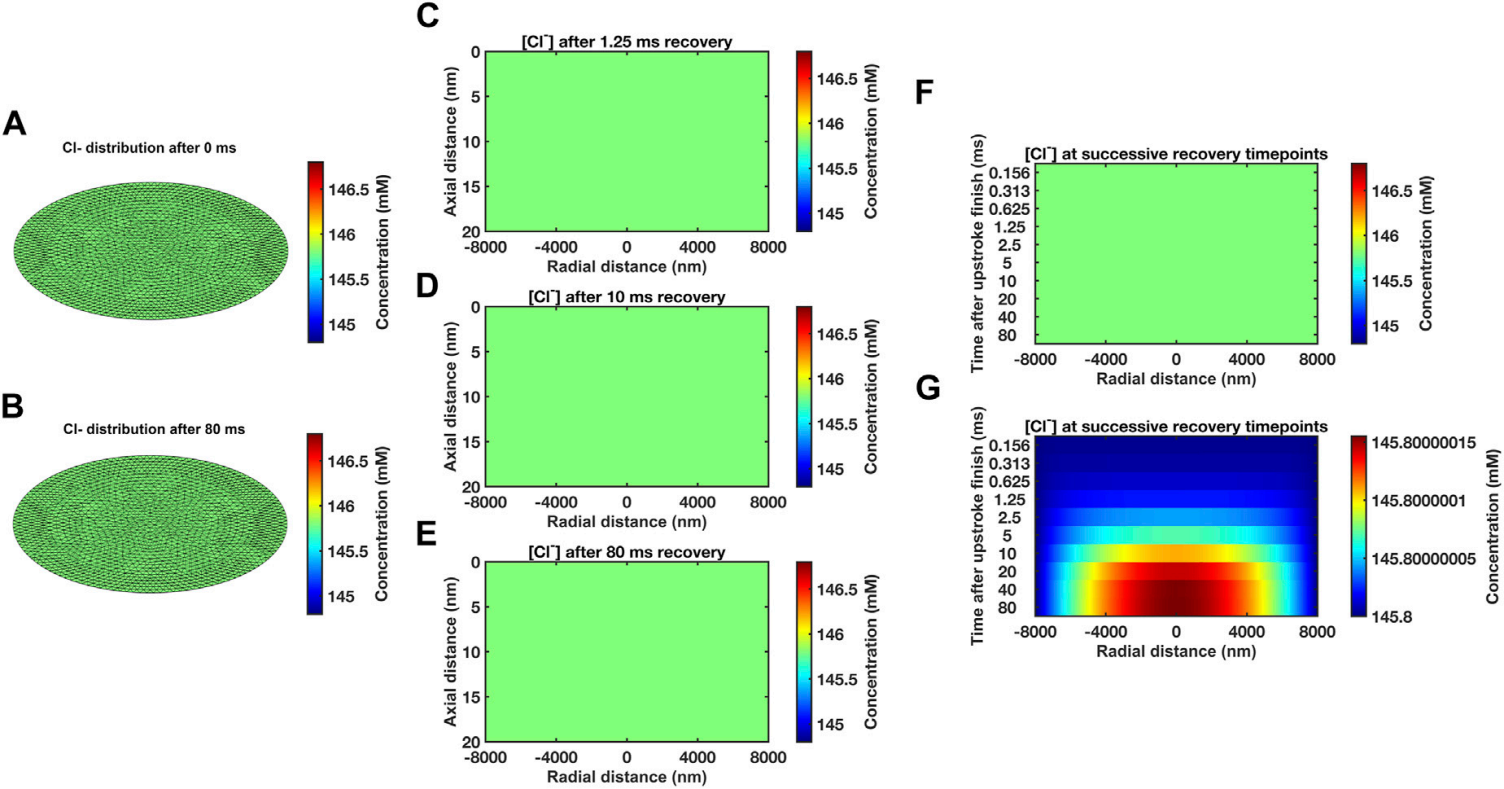
*Temporal and spatial [Cl* ^*−*^ *] gradients through atrial ephaptic recovery.* **(A, B)** Radial gradients at the outset **(A)** and 80 ms into **(B)** the recovery period. **(C–E)** [Cl^−^] through midline slices taken at successive timepoints (left axis) at the outset **(C)**, 10 ms **(D)** and 80 ms **(E)** into the recovery period. **(F, G)** Sequence of midline slices at successive time points through the recovery period (left axis): **(F)** uses the same colour scale as **(A–E)**. **(G)** uses an expanded colour range to permit demonstration of small changes in [Cl^−^] distribution.

Significant [Cl^−^] changes were contrastingly absent whether at the beginning (Figures 5A,C), in the course of (Figure 5D), or at the end of the recovery intervals (Figures 5B,E), whether in the radial direction (Figures 5A, B) or along axial midline sections (Figures 5C–E). Successive heatmaps systematically representing radial and axial [Cl^−^] distributions through the timecourse of the recovery confirmed these findings (Figure 5F). Use of expanded colour scales focussing on changes in rather than absolute concentration indicated [Cl^−^] changes <1.5 × 10^−7^ mM, of <0.0000001% (Figure 5G), around otherwise uniformly stable ∼154.8 mM concentrations, through the present and all subsequent computations.

Evaluations of the resolution and stability of the computational solutions and their underlying mathematical functions under the chosen simulation conditions examined the effects on these of altered stepsize involving both mesh sizes and stepsize intervals. These were varied from the initially selected 0.01 ms stepsize interval and 400 nm mesh size used above (Supplementary Table S1). First, mesh size was successively varied at a fixed 0.01 ms stepsize interval, extending to a fourfold increased, 1,600 nm, mesh size. Secondly, stepsize interval was varied with fixed 400 nm and then with a substantially increased 1,200 nm mesh size. In the latter case, the range of stepsize intervals was extended to the range 0.005–0.08 ms. All these manoeuvres yielded concordant results, exemplified in results from employing the widest mesh size = 1,200 nm and stepsize interval = 0.08 ms (Supplementary Figure S2).

### 4.3 Electrodiffusive recovery in the ventricular ephaptic space

**FIGURE 6:**
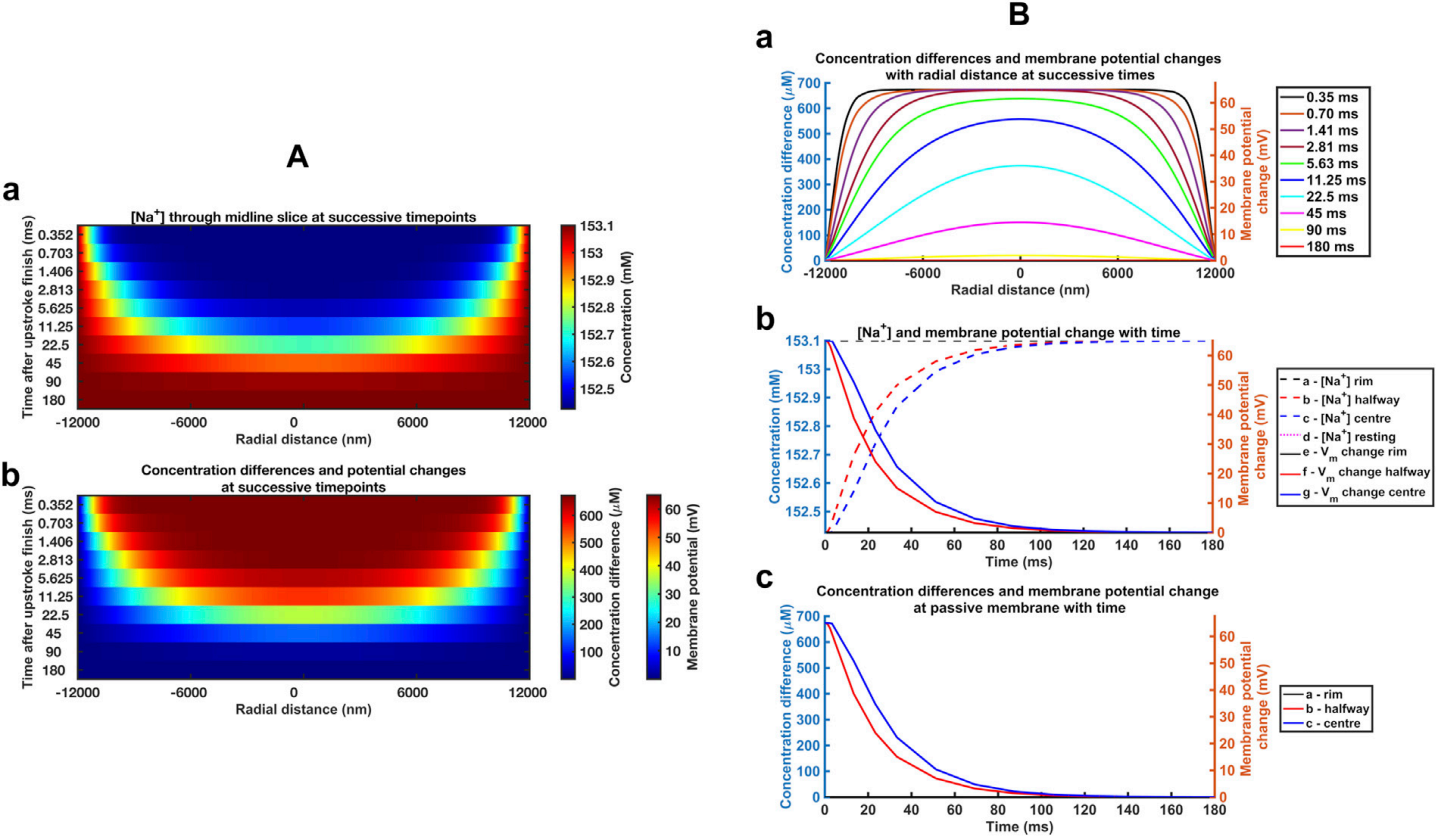
*Time and spatial dependences of the ventricular ephaptic recovery process.* **(A)** Midline slice heat maps at successive time points of recovery representing **(a)** [Na^+^] and **(b)** ionic concentration differences and membrane potential changes. **(B)** Quantification of the temporal and spatial recovery: **(a)** [Na^+^] spatial profiles with time and **(b, c)** recovery timecourses of **(b)** [Na^+^] and membrane potential, and **(c)** ionic concentration differences at the ephaptic rim, ephaptic centre and half-way between the two, respectively.

Figure 6 displays corresponding midline slice [Na^+^] (Figure 6Aa), ionic charge concentration differences (Figure 6Ab), corresponding membrane potential changes (Figure 6Ab), and their spatial profiles at successive logarithmically incremented time points during recovery in a ventricular ephaptic space of 12,000 nm radius (Severs, 2000). These similarly yielded absolute values and spatial profiles of spatial [Na^+^] and membrane potential profiles (Figure 6Ba). The quantification also yielded detailed monotonic recovery timecourses of the [Na^+^] and membrane potential changes at the ephaptic rim, ephaptic centre and half-way between the two (Figure 6Bb). They also compared the ionic concentration differences with the membrane potential change with time (Figure 6Bc). The recovery trends were similar to that of the atrial ephaptic space but extending over increased 180 ms recovery periods.

### 4.4 Geometrical and diffusional effects on ephaptic recovery

**FIGURE 7:**
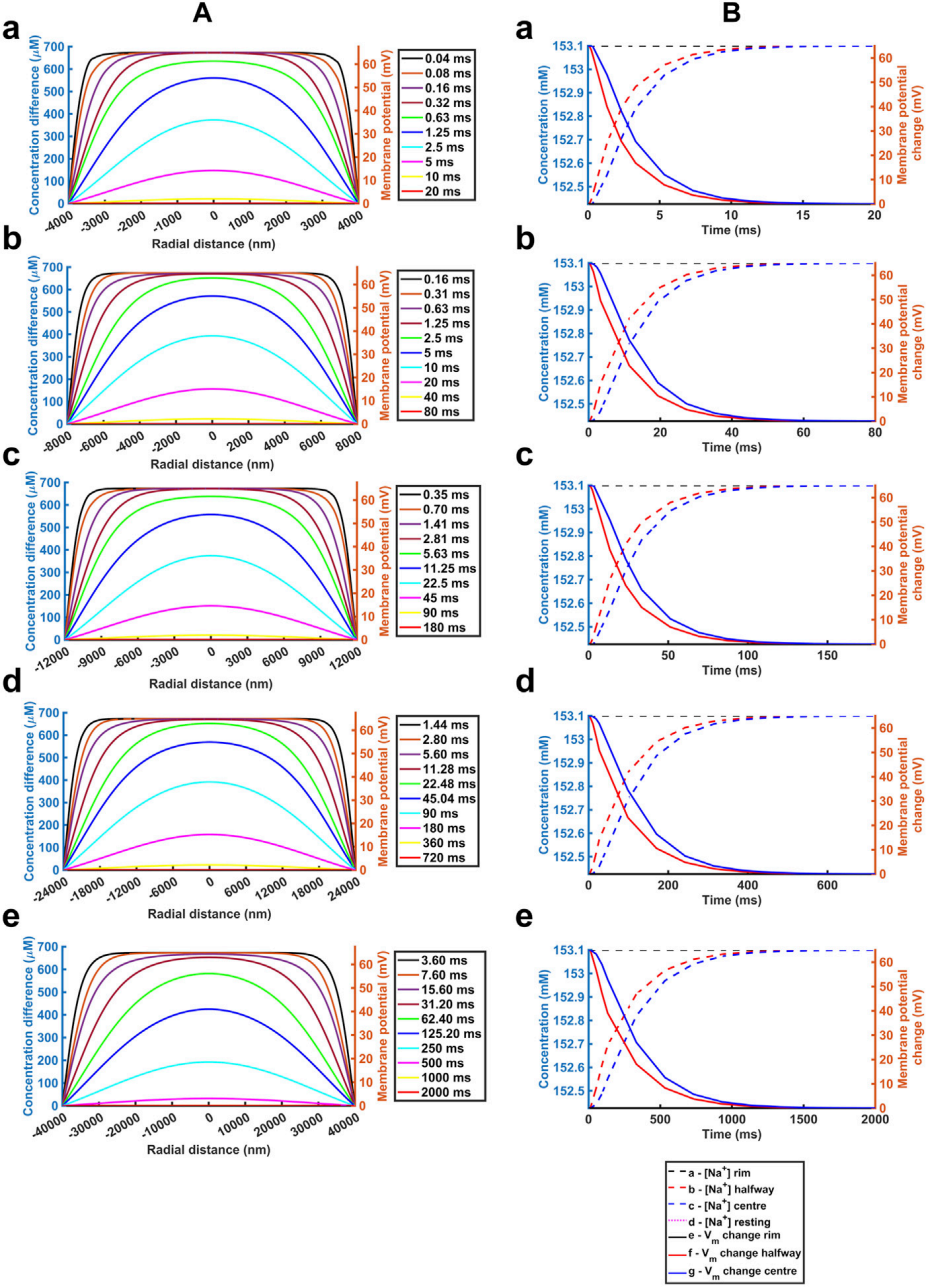
*Quantification of temporal and spatial ephaptic recovery at varying a and constant w, D*_*Na*_ *and D*_*Cl*_. **(A)** [Na^+^] spatial profiles with time; **(B)** Recovery timecourses of [Na^+^] and membrane potential and ionic concentration differences at the ephaptic rim, ephaptic centre and half-way between the two. Values of *a* varied through *a* = 4000 **(a)**, 8000 **(b)**, 12000 **(c)**, 24000 **(d)** and 40000 nm **(e)** respectively.

We next explored the effects of further variations involving the important biological parameters, the ephaptic radius, *a*, the axial distance, *w*, and the relevant Na^+^ and Cl^−^ diffusion coefficients, *D*_Na_ and *D*_Cl_ (Table 2) The computations adopted stepsize all falling within the range validated above. A 0.01 ms stepsize interval and 400 and 800 nm mesh sizes were used when modelling the respective atrial and ventricular parameters, with larger stepsizes when modelling purkinje cell properties. First, varying *a* above and below reported atrial and ventricular radii modelled above explored the sensitivities of the solutions to decreasing or increasing ephaptic radii representing either varying actual ephaptic radii or the effects of membrane folding in the intercalated disk. The latter extended to ephaptic junctions between purkinje cells completing the data set for different cardiac cell types. The computations completed a data set of results of halving the atrial, doubling the ventricular ephaptic space and finally including the purkinje ephaptic space. This completed a range of radii, *a*, of 4,000 nm, 8,000 nm, 12,000 nm, 24,000 nm, and 40,000 nm respectively. In modelling the purkinje ephaptic space 0.4 and 0.8 ms stepsize intervals yielded indistinguishable results. Figure 7 demonstrates times required to return to 99.99975% of resting [Na^+^] and 99.94% and 99.95% at the ephaptic centre respectively. These were, in order of increasing ephaptic radius, 20, 80, 180, 720 and 2000 ms. They fell along a function suggesting that recovery time was directly proportional to the square of the ephaptic radius (*a* ^2^).

**TABLE 2:** Effects of ephaptic geometry and diffusion properties on ephaptic recovery times.

Ephaptic geometry		Diffusion parameters		Computational parameters			Computational results
Ephaptic radius, *a* nm	Axial distance, *w*, nm	Na^+^ diffusion coefficient, *D*_Na_, nm^2^.s^-1^	Cl^−^ diffusion coefficient, *D*_Cl_, nm^2^.s^-1^	Mesh size, nm	Stepsize interval, ms	Computation duration, ms	Time taken for 99.97% recovery ms
4,000	20	1.3 × 10^9^	2 × 10^9^	400	0.01		20
8,000^a^				400	0.01		80
12,000^b^				800	0.01		180
24,000				1,200	0.08		720
40,000^c^				1,600	0.40		2000
8,000	5	1.3 × 10^9^	2 × 10^9^	400	0.01		80
	10			400	0.01		80
	20			400	0.01		80
	40			400	0.01		80
12,000	5	1.3 × 10^9^	2 × 10^9^	800	0.02		180
	10			800	0.02		180
	20			800	0.01		180
	40			800	0.01		180
8,000	20	0.65 × 10^9^	1.0 × 10^9^	400	0.01		160
		1.3 × 10^9^	2 × 10^9^	400	0.01		80
		2.6 × 10^9^	4 × 10^9^	800	0.01		40
12,000	20	0.65 × 10^9^	1.0 × 10^9^	800	0.02		360
		1.3 × 10^9^	2 × 10^9^	800	0.01		180
		2.6 × 10^9^	4 × 10^9^	800	0.01		90

a-c: diameters of atrial^a^, ventricular^b^, and purkinje cell^c^, cardiomyocytes.

Secondly, previous reports had suggested possible functional roles of a fixed *w* term resulting from mechanical couplings between opposing Nav1.5β subunits in the respective active and passive membranes (Veeraraghavan et al., 2018; Salvage et al., 2020a). We accordingly modelled atrial (Supplementary Figure S3) and ventricular (Supplementary Figure S4) junctions with axial distances of *w* = 5, 10 nm and 40 nm respectively, comparing results with those from the control axial distance *w* = 20 nm. Supplementary Figure S3 demonstrates absolute [Na^+^] varying with *w*, reflecting the constant initial molar Na^+^ transfer from the ephaptic volume. For example, doubling the axial distance halved the ionic concentration differences at any defined spatio-temporal point. Nevertheless, trends in the spatial and temporal [Na^+^] recovery profiles were proportionally similar, and the corresponding absolute voltage recovery profiles were indistinguishable between different values of *w*. Hence ephaptic recovery is not greatly affected by the changes in axial distance explored.

Thirdly, varying the diffusion coefficients could assess for the effects on any restrictions to diffusion attributable to the presence of proteins in the ephaptic space, and non-uniformities in *w* that might arise from regions where the component ephaptic membranes are more closely associated as at gap junction plaques. We explored the effects of halving and doubling *D*_Na_ and *D*_Cl_ in the presence of the adopted atrial (Supplementary Figure S5) and ventricular (Supplementary Figure S6) ephaptic space parameters of *a* and *w*. Supplementary Figure S5,6 demonstrate similar initial conditions; these were followed by [Na^+^] and voltage recoveries. However, their time periods varied through values of 160, 80 and 40 ms in the atria and 360, 180 and 90 ms in the ventricle for the runs with halved, normal and doubled *D*_Na_ and *D*_Cl_. Hence the time-period for recovery is affected by the relative value of the diffusion coefficients with a doubling of these halving the recovery time-period for recovery. Furthermore, equivalent time points showed similar patterns of radial recovery. For example, 1.25 ms within an 80 ms recovery period gave a similar recovery as at 2.5 ms out of an 160 ms recovery period.

Table 2 summarises the results from the simulations completed here, providing recovery time data for comparison with reported cardiac cycle lengths made in the Discussion. They predicted ephaptic recovery times of order 40 and 180 ms in atrial and ventricular cardiomyocytes. High limits from doubling ventricular radius or halving diffusion coefficient gave respective 720 and 360 ms recovery times. Finally, the larger diameter purkinje fibres gave recovery times of order 2000 ms.

### 4.5 Dependences of recovery half times upon *a*, *w* and *D*_Na_ and *D*_Cl_

**FIGURE 8:**
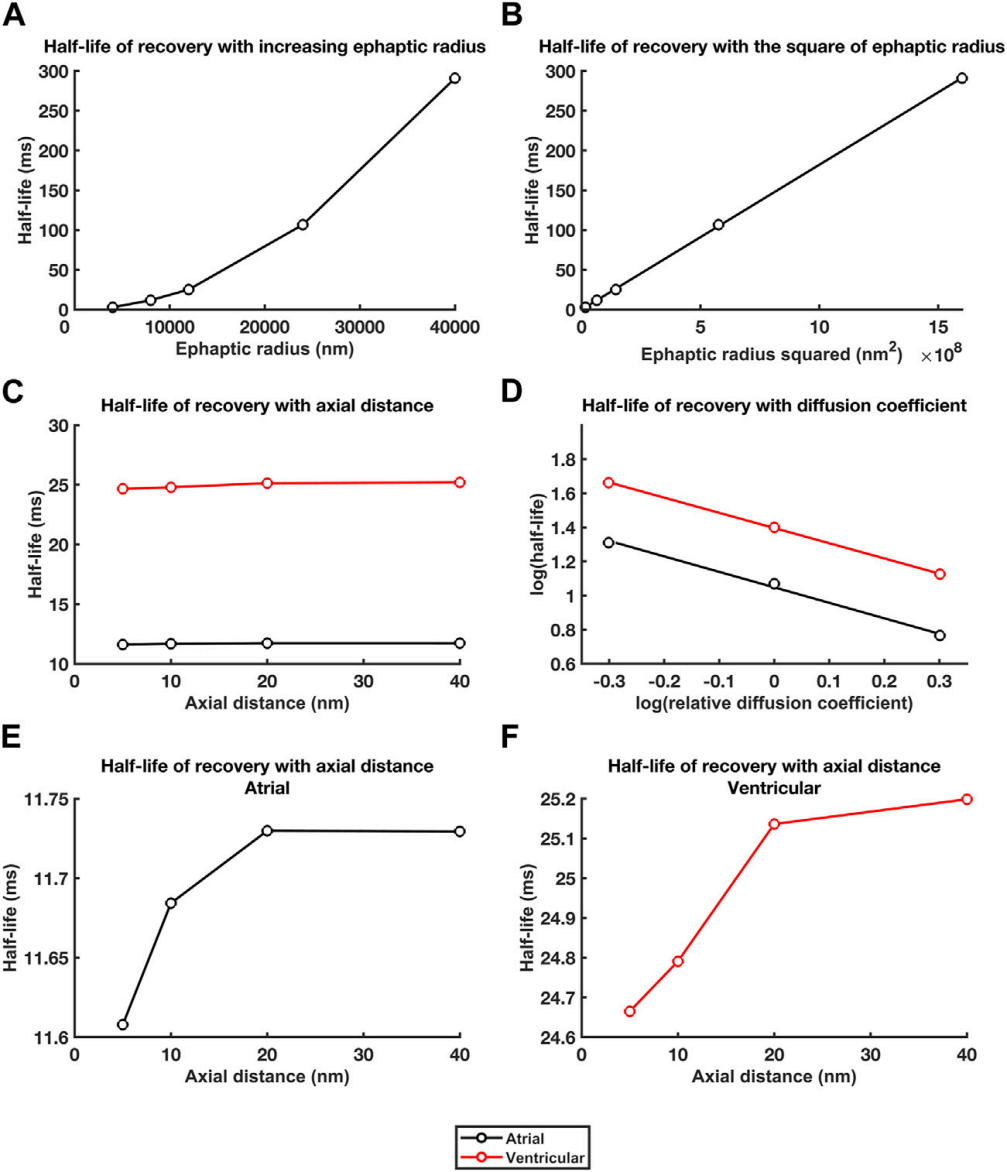
*Dependences of recovery half times upon a, w and D*_*Na*_ *and D*_*Cl*_. Relationships between recovery half times and: **(A, B)** ephaptic radius, *a* **(A)**, and *a* ^2^ **(B)**, **(C)** atrial (black lines) and ventricular (red lines) axial distance, *w*, and **(D)** in double logarithmic plots, relative diffusion coefficients. **(E, F)** Small trends in atrial **(E)** and ventricular recovery half times **(F)** with axial distance, w, following magnification of the ordinate.

Figure 8 summarises these effects in terms of the dependences of recovery half times, $t_{1/2}$ , upon *a*, *w* and *D*_Na_ and *D*_Cl_ respectively. The computations were consistently performed with a 800 nm mesh size and stepsize intervals of 0.01 and 0.02 ms for the atrial and ventricular ephaptic spaces respectively. Results were in agreement with those using 1,200 nm and 0.08 ms mesh sizes used to study the purkinje cell ephaptic space. This first demonstrated relationships between *t*_1/2_, upon *a* (Figure 8A) that yielded a linear *t*_1/2_, - *a* ^2^ relationship (Figure 8B). Secondly, *t*_1/2_ showed little variation with values of *w* on the millisecond scale (Figure 8C), although there were sub-millisecond effects of increasing *w* that appeared to plateau around *w* > 20 nm (Figures 8E,F). Finally, the linear double logarithmic plots of *t*_1/2_ against the relative diffusion coefficients (Figure 8D) yielded lines of best fit of the form:

$$\log\left(t_{1/2}\right)\;=m\;\log\left(relative\;diffusion\;coefficient\right)+c$$ (33)

For the atrial data, determination of the constants *m* and *c*, gave:

$$\log\left(t_{1/2}\right)\;=1.1546\;-\;1.1013\times \log\left(relative\;diffusion\;coefficient\right)$$ (34)

For which,

$$t_{1/2}\;=14.2758\times {\left(relative\;diffusion\;coefficient\right)}^{-1.1013}$$ (35)

For the ventricular data,

$$\log\left(t_{1/2}\right)\;=1.5665\;-\;1.1220\times \log\;\left(relative\;diffusion\;coefficient\right)$$ (36)

For which,

$$t_{1/2}=36.8553\;\times {\left(relative\;diffusion\;coefficient\right)}^{-1.1220}$$ (37)

## 5 Discussion

### 5.1 Gap junction and ephaptic conduction of the cardiac action potential

Conduction of cardiac myocardial excitation involves action potential (AP) generation in active cardiomyocytes electrotonically depolarising membranes of adjacent, initially quiescent, cells within the syncytium, thereby activating their Nav1.5 Na^+^ channels. Additional to gap junction mediated electrotonic current flow (Rohr, 2004), increasing evidence implicates ephaptic excitation in ***activation*** of such conduction (Sperelakis and Mann, 1977; Carmeliet, 2019). Ephaptic conduction has a widespread occurrence; specific examples transmit either bidirectional or unidirectional cell-cell excitation (Furshpan and Potter, 1959) alone (Watanabe and Grundfest, 1961; Bennett, 1977) or combined with other electrical or chemical mechanisms (Rash et al., 1996). In the heart, it could occur at specialised perinexal regions separating adjacent cardiomyocytes (Rhett and Gourdie, 2012; Rhett et al., 2013; Veeraraghavan et al., 2014). It could either complement, or in certain pathological circumstances replace, conduction through well-established local circuit, gap junction (GJ) current pathways (Rohr, 2004) involving ventricular connexin, Cx43 and atrial, Cx43 and Cx40 (Verheule et al., 1997). Syncytial myocardial conduction may then persist despite compromised gap junction coupling following clinical ischaemic insult (Rohr, 2004), atrial fibrillation (Chaldoupi et al., 2009) and in experimental loss-of-function, Cx43 (Eloff et al., 2001; Gutstein et al., 2001) or Cx40, genetic platforms (Hagendorff et al., 1999; Bagwe et al., 2005).

### 5.2 Ephaptic activation of conduction between adjacent cardiomyocytes

Existence of ephaptic ***activation*** of conduction between cardiomyocytes has both experimental and theoretical support (Léonetti, 1998; Koch, 2004; Moise et al., 2021). Electron microscopy of perinexal region specialisations demonstrated closely apposed adjacent component cell membranes (Veeraraghavan et al., 2015) with increased Na_v_1.5 in addition to Cx43 expression. Super-resolution microscopy demonstrated dense Nav1.5 clusters within 200 nm of GJ plaques (Veeraraghavan et al., 2015; Hichri et al., 2018; Salvage et al., 2020b). Smart patch clamping indicated elevated junctional over nonjunctional *I*_Na_ (Veeraraghavan et al., 2018). Elevations in perinexal β1 (SCN1B) expression, co-localised with that of the Nav1.5 potentially providing adhesion *trans* scaffolds ensuring narrowed (∼20 nm) perinexal clefts adjacent to the gap junctions (Veeraraghavan et al., 2018; Salvage et al., 2020a). βadp1 peptide inhibited this β1-mediated adhesion, widened guineapig ventricular perinexi, reduced perinexal but not whole cell *I*_Na_ and pro-arrhythmically slowed AP conduction (Veeraraghavan et al., 2018).

Mathematical modelling of linear strands of cardiac cells under different membrane geometrical, extracellular space, ionic concentration and diffusion conditions suggested that AP propagation can persist even with compromised GJ coupling (Sperelakis and Mann, 1977; Sperelakis, 2002; Mori et al., 2008). They also raised the possibility of alternating ephaptic and gap-junction-mediated activation (Mori et al., 2008). Such features extended to 3-dimensional myocyte sheets incorporating functioning GJs and more realistic inhomogeneities in extracellular spacing and ionic channel distribution (Lin and Keener, 2010). Recent finite element replications integrated experimentally determined nanoscale transmission electron microscopy intercalated disk structure with measurements of intercellular cleft electrical conductivity. Intermembrane separation and gap junction distribution and their heterogeneity then significantly affected spatial characteristics of electrical polarization within the intercellular cleft modifying the postjunctional Nav1.5 activation (Moise et al., 2021).

### 5.3 Ephaptic recovery following conduction

Fewer studies have examined ***recovery*** processes following ephaptic excitation, essential for any subsequent excitation as in repetitive excitation. The present formalised computational analysis explored limiting electrodiffusive contributions within the ephaptic space, and between ephaptic space and remaining extracellular space to ephaptic membrane recovery for the first time. It then explores effects upon these of ephaptic junction geometrical and diffusional properties. It extends a charge difference approach utilising Gauss’s flux theorem previously introduced to determine membrane voltages across and within arbitrary numbers of compartments separated by defined membrane capacitances (Fraser and Huang, 2004; 2007; Fraser et al., 2011; Pedersen et al., 2011). These are here exemplified by the intracellular compartments of the participating cardiomyocytes, the restricted ephaptic, and the remaining free, extracellular space. This approach determined membrane voltage from *concentration differences* resulting from *recovery fluxes* of *finitely mobile charge* within a *restricted* ephaptic space. It is particularly useful analysing an ephaptic *recovery* involving electrochemically driven ion fluxes producing ion concentration and consequent potential changes dependent upon both compartment volumes and their intervening membrane capacitances. Previous computational modelling contrastingly involved summing of *ionic current* as opposed to *ion concentration* components (Hodgkin and Huxley, 1952; McAllister et al., 1975; Huang and Peachey, 1992; Luo and Rudy, 1994; Puglisi and Bers, 2001). It provides changes in *activation* transmembrane potential differences *between* compartments (Sperelakis, 2002; Mori et al., 2008; Lin and Keener, 2010; Veeraraghavan et al., 2015) but not absolute potentials *within* compartments. This requires at least one compartment to provide a reference potential, and frequently cannot explicitly simulate concentration changes.

### 5.4 Modelling electrodiffusive processes underlying ephaptic recovery

#### 5.4.1 Initiation of electrodiffusive modelling

The present analysis first adopted experimentally established experimental values describing mammalian atrial, ventricular and purkinje cardiomyocyte ephaptic junction anatomy, extracellular, Na^+^ and Cl^−^, electrolyte concentrations and their free diffusion coefficients, *D*_Na_ and *D*_Cl_ (Passiniemi, 1983) and AP upstroke magnitudes (Schoenberg et al., 1975; Severs, 2000; Mori et al., 2008; Richards et al., 2011; Veeraraghavan et al., 2015; 2018). Secondly, computational analysis was initialised by determining a lower limit of transmembrane Na^+^ withdrawal by the AP of the initially active cell by a ΔV∼130 mV AP upstroke at physiological (37°C) temperatures. This would generate domains of reduced [Na^+^] within the restricted diffusional perinexal ephaptic space. This calculation likely provides a lower limit value as it assumes a simple circular geometry of unfolded contiguous membranes of equal radial diameter *a*. Available quantitative information bearing on possible transmembrane fluxes within the ephaptic space does not provide precise experimental values for Nav1.5 channel numbers within the ephaptic space itself. Combining reported peak cardiomyocyte *I*_Na_ = 20–140 pA pF^-1^ ranges (Casini et al., 2009; Rougier et al., 2019) and a ∼120 pF total (guinea-pig) myocyte membrane capacitance (Wan et al., 2003), yields a ∼2,400-16,800 pA range per myocyte. This suggests a typical total 1,200-8,400 Nav channel number per myocyte assuming reported ∼2 pA unit currents reported in neuroblastoma and SCN5A-transfected HEK293 cells (Aldrich and Stevens, 1987; Clatot et al., 2017). Some reports localise ∼50% of cardiomyocyte Na_V_ channels to intercalated disc plasma membranes (Petitprez et al., 2011). This suggests between 600-4,200 active Nav1.5 in each membrane flanking the ephaptic space. A value of 2,400 channels in the middle of this range for atrial cardiomyocytes gives a 4,800 pA = 4,800 × 10^−12^ C s^-1^ current at the intercalated disk. The resulting $4.9748\times \;10^{-14}\;\text{mol }s^{-1}$ molar Na^+^ transfer rate predicts a 3.4824 × 10^−17^ mol Na^+^ removal from the ephaptic space during the action potential upstroke assuming ∼0.7 ms mean Na^+^ channel open times (Sigworth and Neher, 1980). This exceeds the limiting values adopted here.

#### 5.4.2 Modelling the electrodiffusive recovery

Thirdly, finite element computation utilising three-dimensional Nernst-Planck electrostatic equations determined the extracellular Na^+^ and Cl^−^ electrodiffusional fluxes. This yielded the time-dependence of axial and radial ionic concentrations within the three-dimensional ephaptic space both immediately following the AP and its subsequent recovery (Léonetti, 1998; Koch, 2004). Computations of the timecourse and spatial distribution of the resulting [Na^+^] changes adopted a bilaterally and radially symmetrical atrial ephaptic space flanked by uniformly contiguous directly opposing membranes of equal area and specific capacitance, corresponding to equal total capacitances (Schoenberg et al., 1975; Severs, 2000; Mori et al., 2008; Richards et al., 2011). Immediately following Na^+^ transfer, radial [Na^+^] gradients were absent in [Na^+^] heat maps. Axial gradients in [Na^+^] and charged ion concentration differences were detectable only at high colour magnifications. Such conditions were confirmed in computations obtained over the first millisecond of recovery.

Fourthly, the consequent membrane potential changes induced by the Na^+^ withdrawal were then approximated applying Gauss’s Flux theorem. The majority of the electric flux, $\Phi_{Ê}$ resulting from the resulting charging of the ephaptic space would traverse its bounding membranes. Thus, these two closely apposed, active and passive, membranes were separated by a small gap distance *w* << *a*. Hence membrane areas accounted for most of the gaussian surface surrounding the ephaptic space in comparison to its rim accessing the extracellular compartment: π*a* ^2^>>2π*ad*. The simulations then sought to determine voltage change ∆V in the passive membrane and its subsequent recovery, produced by AP activation in the active membrane of the ephaptic junction.

For an ephaptic space of uniform radial extent comprising closely apposed participating membranes each of equal area or total capacitance, the latter membrane would fire an AP were ∆V to exceed the threshold amplitude, ∆V> Δ*V*_th_,. More general treatments for the latter firing condition could consider passive and active opposing membranes of differing surface areas, *S*th and *S*_AP_ respectively, and similar *C*_m_. An action potential, amplitude Δ*V*_AP,_ would now transfer from the ephaptic space, total charge,

$$q=S_{\text{AP}}C_{m}{∆V}_{\text{AP}}$$ (38)

In the limit corresponding to small gap distance *w*→ 0, the two membranes will show voltage change following the excitation condition, illustrating the importance of the relative areas of the active and passive membranes, and their implications for a capacity for uni- or bi-directional conduction:

$$\Delta V=\frac{S_{\text{AP}}{∆V}_{\text{AP}}}{S_{\text{AP}}+S_{\text{th}}}>\;{∆V}_{\text{th}}$$ (39)

Characterisation of the subsequent recovery phase simulated Na^+^ diffusion from the remaining extracellular space into the radially symmetrical atrial cardiomyocyte ephaptic space. Communication of ephaptic space with a well stirred extracellular fluid was reflected in Dirichlet boundary conditions holding constant the ephaptic rim to established, 153.1 mM [Na^+^] and 154.8 mM [Cl^−^], extracellular values (Trautwein et al., 1962; Dijkman et al., 1997; Lai et al., 2007). The resulting cross sectional and midline sectional heatmaps confirmed uniform, and close to uniform radial [Na^+^] and ion concentration differences at the outset and following recovery. There were uniform axial values with variations in the radial gradients at critical time-points during recovery. Systematic displays and quantified graphs of the recovery timecourses demonstrated marked changes in absolute values and spatial profiles of midline slice [Na^+^], ionic concentration differences and the corresponding membrane potential profiles with time. Their recovery timecourses at the ephaptic rim, ephaptic centre and half-way between the two assumed approximately exponential trends to their final recovered values. In contrast, there were no significant changes in the counterion [Cl^−^] levels through the simulations. Further computations successively varying the parameters of mesh size and stepsize interval holding the remaining parameter constant corroborated these analyses. They demonstrated that all the computations fell within a stepsize parameter space producing consistent and convergent results. Finally, calculations extended to the ventricular ephaptic space demonstrated similar recovery trends that took place over longer recovery periods.

#### 5.4.3 Effects of ephaptic junction anatomy and diffusive properties

These estimations began with considering a simplified and formalised system. However, *in vivo* ephaptic junctions are complex 3-dimensional structures comprising plicate and inter-plicate regions resulting in varying rather than constant ephaptic axial distances (Veeraraghavan et al., 2015; 2018; Vermij et al., 2017). These as well as diffusion restrictions reflecting contained proteins and nonuniformities in ephaptic thickness at gap junction plaques could modify recovery. These complexities would all tend to predict higher initial overall Na^+^ fluxes attributed to AP activation by the active membrane, higher effective radial diameters and lower effective axial distances and Na^+^ and Cl^−^ diffusion coefficients within the ephaptic gaps. All these individually and together would result in more prolonged recovery times than predicted by a formal model, which therefore provide lower limits for such recovery times. Nevertheless, the extent to which these variations might influence the findings was assessed by exploring the effects of systematically increasing and decreasing values of cardiomyocyte radii, axial distances, and adopted Na^+^ and Cl^−^ diffusion coefficient values in turn, whilst the remaining parameters were held constant. These additionally provided useful quantitative insights into the physical consequences of specific properties in the biological systems. These could prompt and guide further detailed experimental study. These assessments first explored the effects of varying ephaptic radius *a*. They thereby assessed the sensitivities of the solutions to ephaptic radius, provided a formal representation of membrane folding in the intercalated disk, and extended the computations to the significantly larger diameter purkinje cells completing the data set for different cardiac cell types. The findings suggested that recovery half-lives were directly proportional to the square of the ephaptic radius. Contrasting results resulted from varying the axial distances *w.* Despite significant effects on absolute [Na^+^] reflecting the constant initial molar Na^+^ transfer from the ephaptic volume, this exerted little significant effect upon voltage recovery profiles. Finally, halving and doubling *D*_Na_ and *D*_Cl_ markedly affected recovery half-lives through double logarithmic relationships.

### 5.5 Physiological significance of the computed properties

Finally, the resulting spatial and temporal electrodiffusion properties of the modelled recovery could be compared with known physiological heart rates, in comparing its contributions to those of the other biomolecules associated with such clusters. Collation of recovery times from these investigations provided limiting indications of the extent to which electrodiffusion processes might contribute to ephaptic recovery in different cardiomyocyte types, pacing conditions, and animal species. The initial modelling conditions predicted ephaptic recovery times of order 40 and 180 ms in atrial and ventricular cardiomyocytes. High limits from doubling ventricular radius or halving diffusion coefficient gave respective 720 and 360 ms recovery times. The larger diameter purkinje fibres gave recovery times of order 2000 ms. All these values could be compared with resting and exercising heart rates amongst mammalian species. For example, resting heart rates of blue whale ∼30; elephant, ∼30; human, ∼70; mouse, ∼650; shrew, ∼835, Etruscan shrew, ∼1,511 bpm, correspond to cardiac cycle durations of ∼2000, ∼2000, ∼850, ∼90, ∼70 and ∼40 ms respectively (Benedict and Lee, 1936; Fons et al., 1997; Ho et al., 2011; Goldbogen et al., 2019; Ponganis, 2021). Electrodiffusion could thus potentially either entirely, or partially but significantly, account for such recovery in atrial and ventricular cardiomyocytes respectively, to extents dependent on the species heart rates.

In contrast, purkinje cardiomyocyte recovery times were clearly incompatible with ephaptic transmission in an absence of mechanisms additional to electrodiffusional processes to aid [Na^+^] recovery within the ephaptic space. Thus, the increased purkinje cell diameter whilst enhancing conduction velocity by increased local circuit gap junction current could compromise ephaptic conduction by reducing its electrodiffusive recovery. Thus, cable theory predicts that conduction velocity attributable to gap junction mediated local circuit currents (King et al., 2013) as opposed to ephaptic conductance contributions depends on membrane capacitance, and cell-cell intracellular resistance, of unit fibre length (see Figure 5 of (Edling et al., 2019)) and that the latter varies with square root of the fibre radius (Huang, 2021). Hence in larger diameter cardiomyocytes, in contrast to their prolonged recovery times *compromising* ephaptic conduction contributions, the resulting increased gap junction conduction positively contributes to action potential propagation velocity. The latter is reflected in the higher conduction velocities shown by larger diameter Purkinje cardiomyocytes. This would contrast with the larger expected relative ephaptic contributions to conduction velocity in smaller diameter atrial fibres.

## 6 Conclusion

In conclusion, the present analysis sought fundamental physical insights into ephaptic conduction and its recovery. It combined electrochemical and Gaussian analyses of diffusive and transmembrane voltage events, in contrast to previous approaches involving circuit modelling solely of electric currents, for a formalised ephaptic space. It initially adopted limiting ephaptic radial diameters, axial distances and Na^+^ and Cl^−^ diffusion coefficients that likely provide lower limiting recovery times. However, it then explored the effects of more complex ephaptic junction organization through systematically varying each of these parameters, to provide more broadly applicable physical insights. These insights could prompt further experimental and theoretical studies scrutinizing the applicability of electrodiffusive recovery to cardiac ephaptic transmission *in vivo* (Struckman et al., 2021). The latter could first determine effects on effective ephaptic radii and diffusion coefficients of membrane plication, nonuniformities in axial distance, diffusive obstructions and local factors affecting diffusion coefficients. One might also speculate that contractile activity could exert mechanical effects upon ephaptic geometry contributing recovery through hydrostatic effects additional to the electrodiffusion processes replacing Na^+^ from the bulk extracellular space analysed here (Salvage et al., 2020a).

Second, the relative contributions of electrodiffusive and actions of other membrane molecules potentially making up a functional ephaptome merit determination. The Nav1.5 occur in clusters within perinexal regions and surround gap junction plaques with large regions of gap junction clusters (Rhett and Gourdie, 2012; Rhett et al., 2013; Gourdie, 2019) ‘pinning’ together the component membranes at discrete sites (Salvage et al., 2020a). These are separated by an ephaptic space permitting diffusion around them. Besides electrodiffusion, ephaptic [Na^+^] or positive charge could also be restored by transport pathways extruding intracellular Na^+^ or other positive charge into the ephaptic space. Confocal microscopy and super resolution studies indicated increased Na^+^-K^+^-ATPase densities (Noël et al., 1991; Mcdonough et al., 1996) particularly around gap and adherence junctions (Struckman et al., 2021), particularly in atrial relative to ventricular myocytes. Cycles of electrogenic intercalated disk Na^+^-K^+^-ATPase activity each would add 3Na^+^ and withdraw 2K^+^ from the ephaptic space (Vermij et al., 2017).

In addition, gated stimulated emission depletion (gSTED) and stochastic optical reconstruction microscopy (STORM) super-resolution microscopy attribute much perinexal K^+^ conductance to Kir2.1 (Difranco et al., 2015; Chen et al., 2018) clustered around desmosomal regions (Veeraraghavan et al., 2016). Their strongly inwardly rectifying property would close depolarised channels, reducing outward current during AP depolarisation, minimising electrical shunting of the Na^+^ transfer. Contrastingly, they would permit positive K^+^ flux into the recovering ephaptic space across repolarised membranes. Thus, acute interstitial oedema-induced perinexal swelling causes a pro-arrhythmic slowed conduction respectively relieved and accentuated by Kir2.1 inhibition and Nav1.5 block (Veeraraghavan et al., 2016).

## Data Availability

The original contributions presented in the study are included in the article/Supplementary Material, further inquiries can be directed to the corresponding author.
