# Supplementary material for: Nernst-Planck-Gaussian modelling of electrodiffusional recovery from ephaptic excitation between mammalian cardiomyocytes
Source: Front Physiol. 2024 Jan 3;14:1280151. doi: 10.3389/fphys.2023.1280151 (PMC10791825; doi:10.3389/fphys.2023.1280151)
Supplement: Supplementary file 1 [file DataSheet1.PDF]

`SUPPLEMENTARY FILE

Contents

A. Results from tests of the Dirichlet condition on midline slice heat maps of concentration and voltage profiles close to the end of the action potential upstroke.

Figure S1. Tests of the Dirichlet condition on midline slice heat maps of concentration and voltage profiles close to the end of the action potential upstroke

B. List of computational runs testing differing mesh sizes and sampling intervals

Table S1. Finite intervals explored for assessing computational analysis
Figure S2. Computational runs testing differing mesh sizes and sampling intervals.

C. Supplementary figures for Results

D. MATLAB Code

SPECIFY PARAMETERS
Geometry
Boundary Conditions
Constants
Timings
MODEL PROGRAMMING
Geometry and Mesh
Coefficients
Boundary and Initial Conditions
SOLUTION
Run model
DATA PRESENTATION
Heat Maps
Time-Dependent Recovery Graphs
Spatio-Temporal Recovery Graph
Midline Slice Plot
Midline Slice Plots with Times

A. *Tests of the Dirichlet condition on midline slice heat maps of concentration and voltage profiles close to the end of the action potential upstroke.*

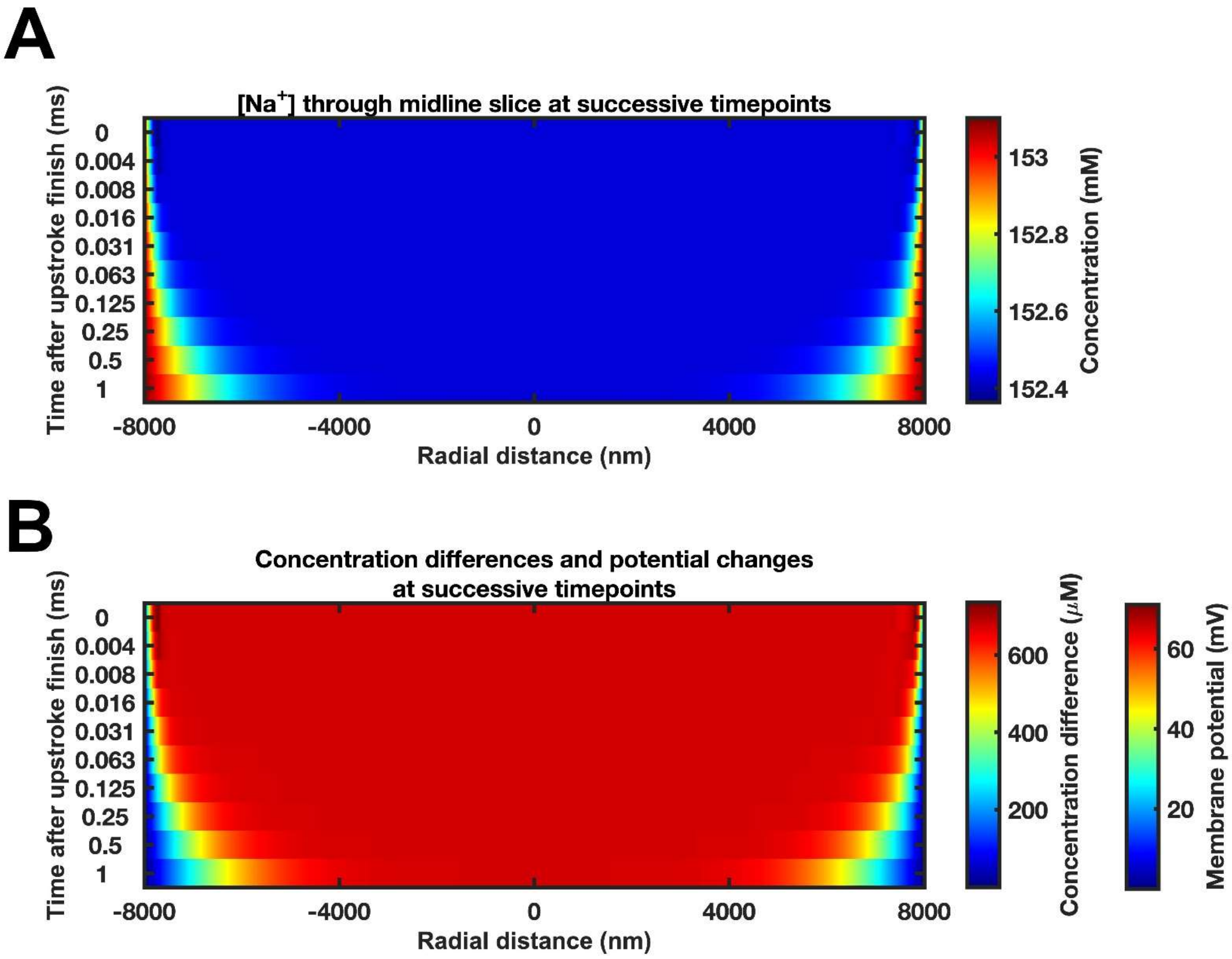

Figure S1. Tests of the Dirichlet condition on midline slice heat maps of concentration and voltage profiles close to the end of the action potential upstroke. For comparison with Figure 2. Series of midline slice heat maps at successive time points of recovery (left axis) over the first millisecond of recovery beginning from the initial conditions derived from the established Na^+ transfer. (A) [Na^+] and (B) ionic concentration differences. Scale bars on right: concentration differences (A, B) and membrane potential changes (B) at the passive membrane. Mesh size 400 nm; sampling interval 0.001 ms. Simulation of the early recovery process assuming uniform concentration profiles for the initial conditions, and ephaptic rim assigned Dirichlet boundary conditions holding constant [Na^+] = 153.1 mM and and [Cl^-] = 154.8 mM representing a well stirred bulk extracellular fluid. Such simulations indeed yielded demonstrated indistinguishable results. Note that applying the Dirichlet boundary conditions at the ephaptic rim produced a singularity in radial concentration over ~350 nm at the ephaptic rim. However, the figure demonstrates its rapid resolution at the start of the recovery period, in which the initially infinite first derivative at the boundary apparent at 0 ms and 0.004 ms is resolved by 0.008 ms.

B. List of computational runs testing differing mesh sizes and sampling intervals

Table S1. Finite intervals explored for assessing computational analysis

(1) Examining the effect of mesh size at fixed sampling interval

At sampling interval: **0.01** ms:
Mesh sizes: 325, **400**, 800, **1200**, 1600 nm

(2) Examining the effect of sampling interval at fixed mesh size

At mesh size: **400** nm
Sampling intervals: 0.00615, **0.01**, 0.02 ms

At mesh size: **1200** nm
Sampling intervals: 0.005, 0.00615, **0.01**, 0.02., 0.04, 0.08 ms

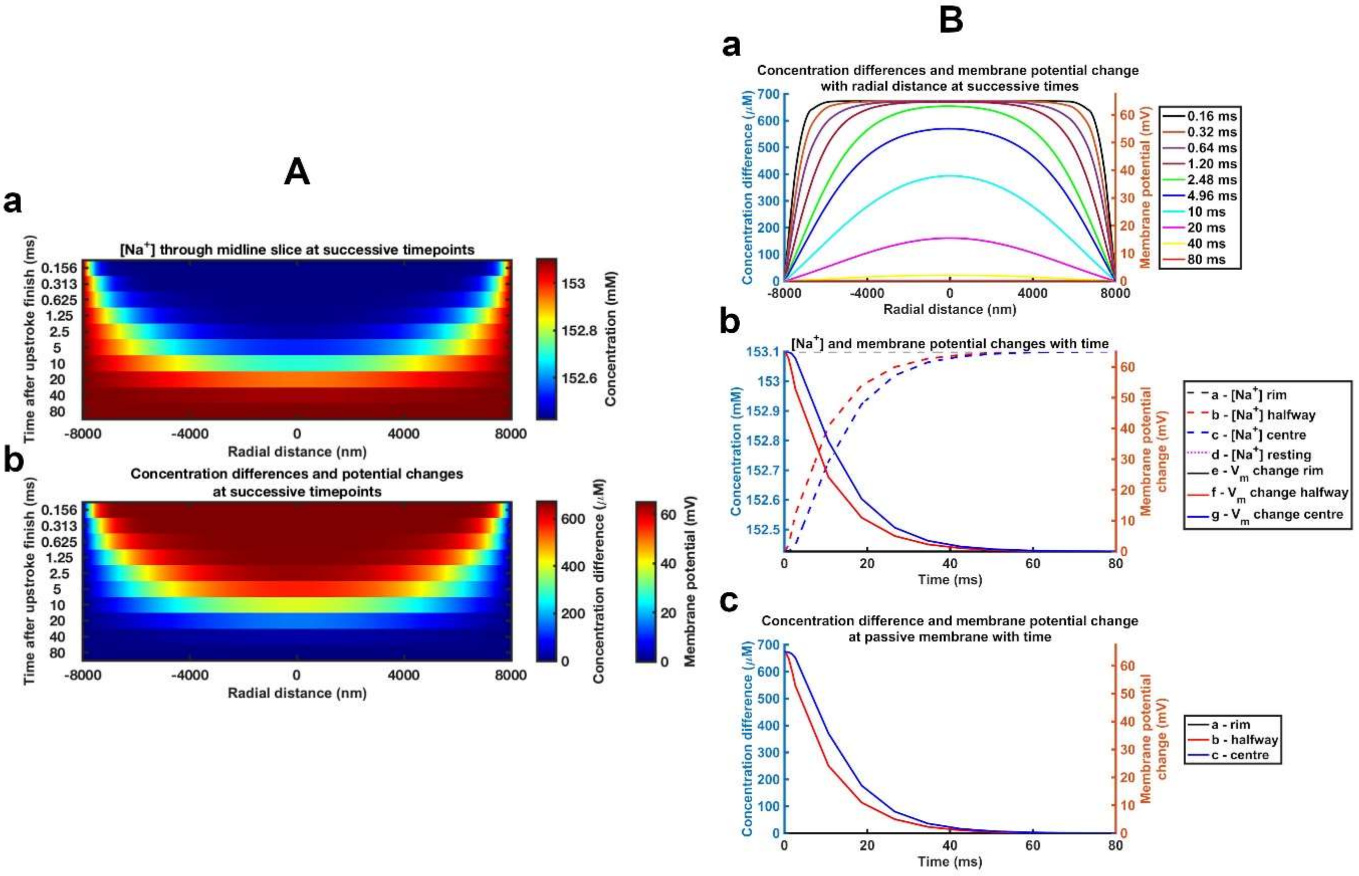

Figure S2. Computational runs testing differing mesh sizes and sampling intervals. The extreme case employing mesh size = 1200 nm and sampling interval = 0.08 ms. Note agreement with results shown in Figure 4. (A) Midline slice heat maps at successive time points of recovery representing (a) $[Na^+]$ and (b) ionic concentration differences and membrane potential changes. (B) Quantification of the temporal and spatial recovery. (a) $[Na^+]$ spatial profiles with time. (b,c) Recovery timecourses of (b) $[Na^+]$ and membrane potential and (c) ionic concentration differences respectively at the ephaptic rim, ephaptic centre and half-way between the two.

C. Supplementary figures for Results

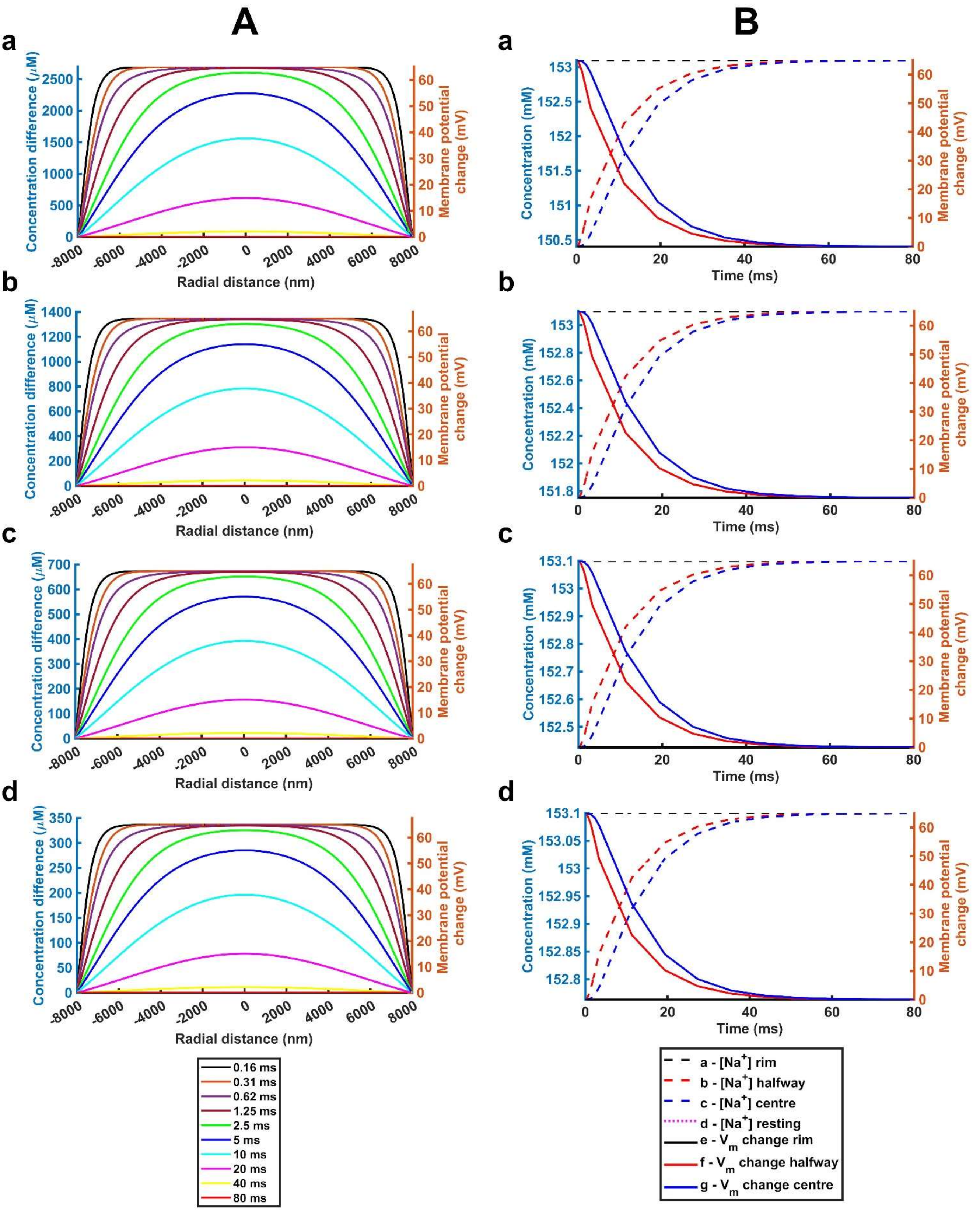

Supplementary Fig. S3. ***Quantification of atrial temporal and spatial ephaptic recovery at varying w and constant a, D_{Na} and D_{Cl}.*** (A) [Na^+] spatial profiles with time; (B) Recovery timecourses of [Na^+] and membrane potential and ionic concentration differences at the ephaptic rim, ephaptic centre and half-way between the two. Values of *w* varied through *w* = 5 (a), 10 (b), 20 (c) and 40 nm (d) respectively.

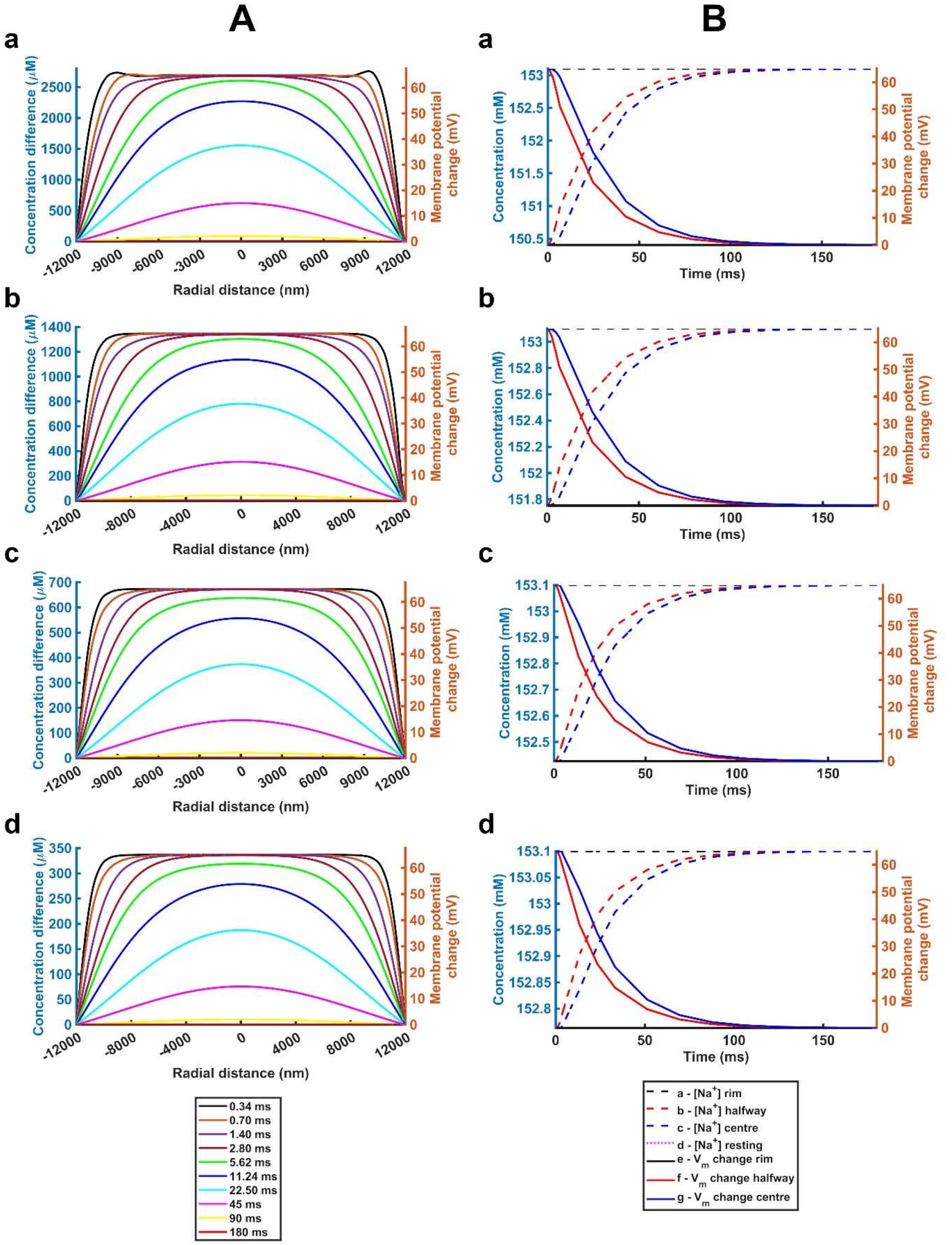

Supplementary Fig. S4. ***Quantification of ventricular temporal and spatial ephaptic recovery at varying w and constant a, D_{Na} and D_{Cl}.*** (A) [Na^+] spatial profiles with time; (B) Recovery timecourses of [Na^+] and membrane potential and ionic concentration differences at the ephaptic rim, ephaptic centre and half-way between the two. Values of *w* varied through *w* = 5 (a), 10 (b), 20 (c) and 40 nm (d) respectively.

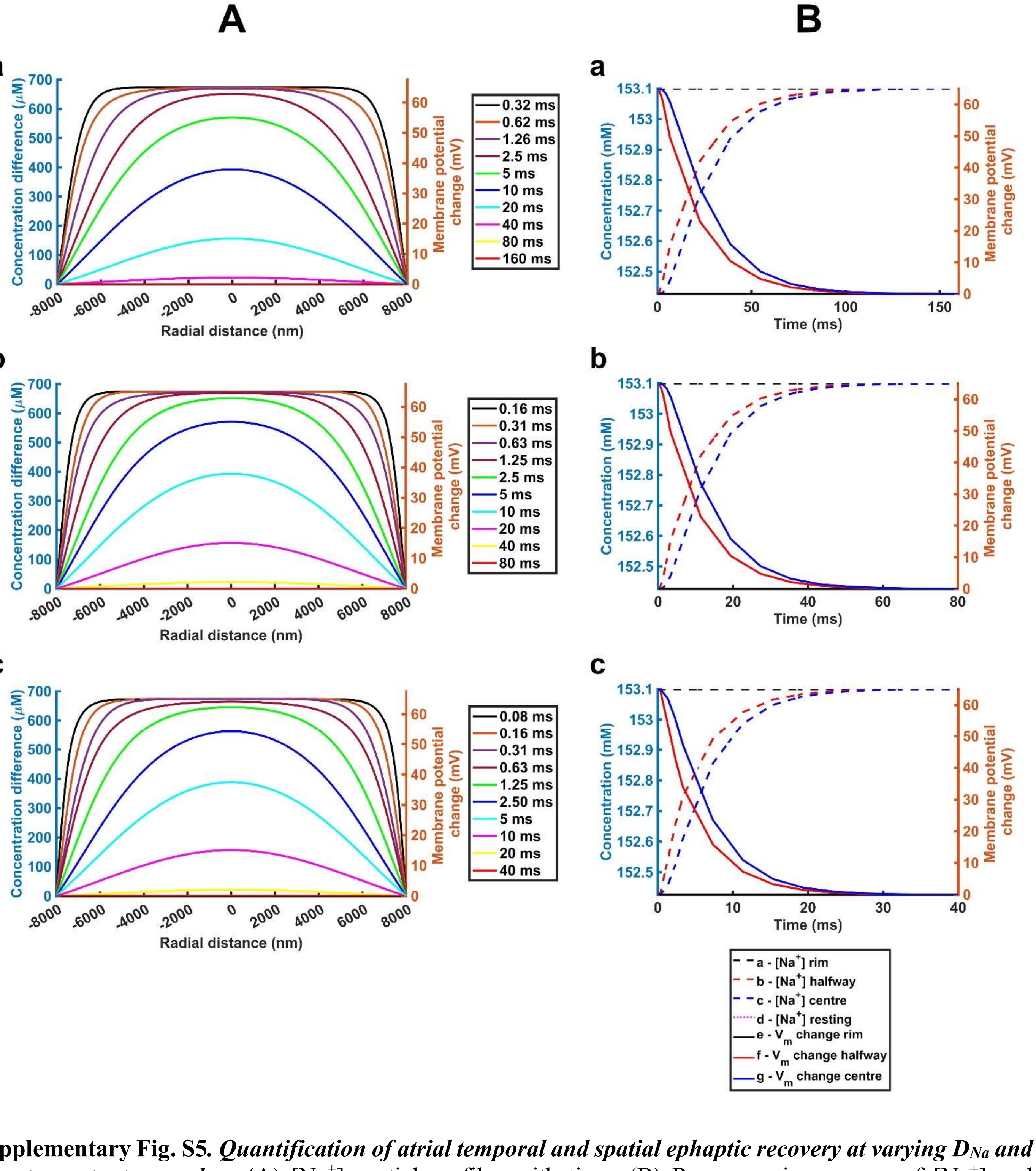

Supplementary Fig. S5. ***Quantification of atrial temporal and spatial ephaptic recovery at varying D_{Na} and D_{Cl} at constant a, and w.*** (A) [Na^+] spatial profiles with time; (B) Recovery timecourses of [Na^+] and membrane potential and ionic concentration differences at the ephaptic rim, ephaptic centre and half-way between the two. Values of (D_{Na}, D_{Cl}) varied through (D_{Na}, D_{Cl}) = (0.65×10^9, 1.0×10^9) (a), (1.3×10^9, 2×10^9) (b) and (2.6×10^9, 4×10^9) $nm^2.s^{-1}$ (c) respectively.

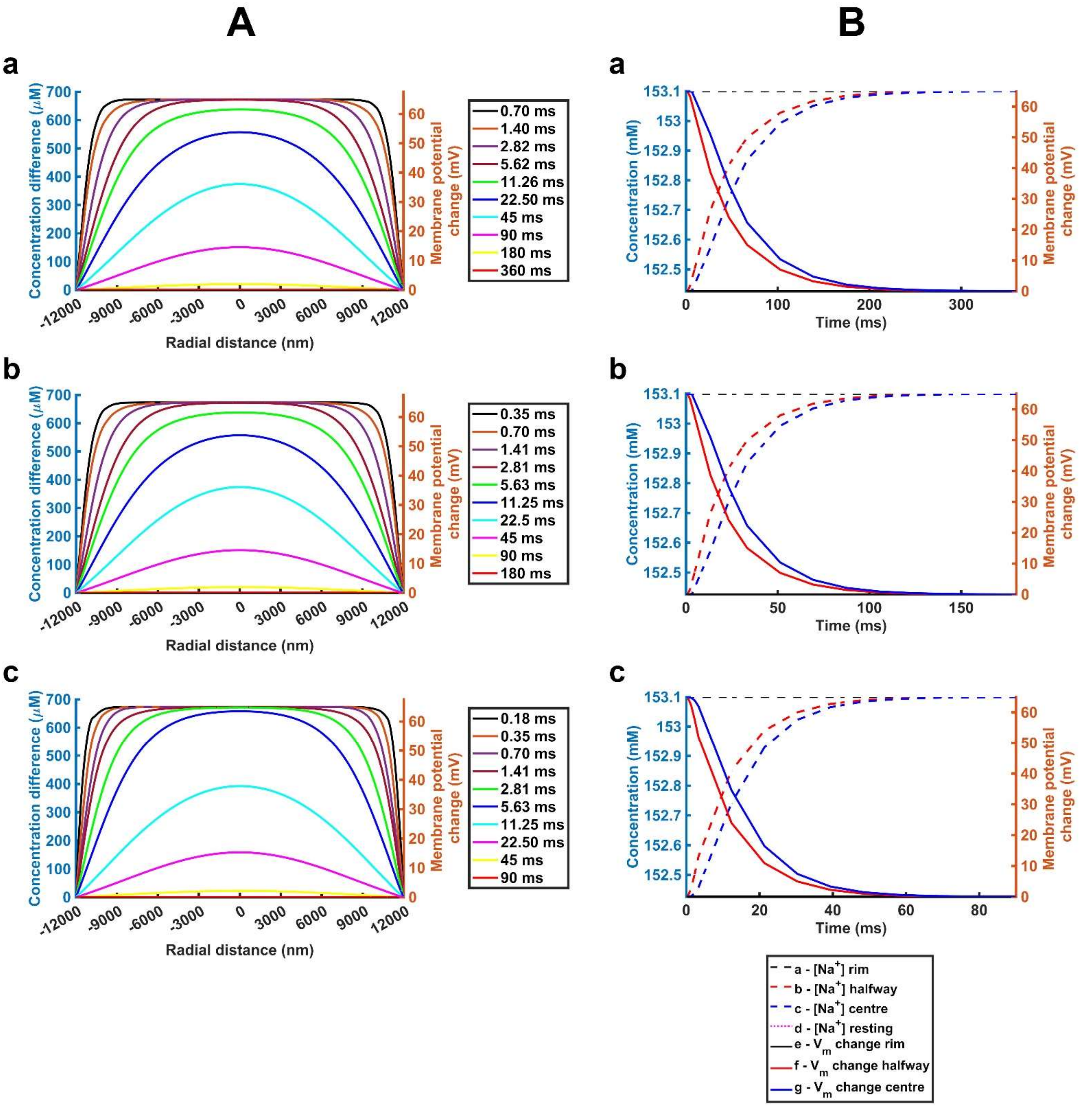

Supplementary Fig. S6. ***Quantification of ventricular temporal and spatial ephaptic recovery at varying D_{Na} and D_{Cl} at constant a, and w.*** (A) [Na^+] spatial profiles with time; (B) Recovery timecourses of [Na^+] and membrane potential and ionic concentration differences at the ephaptic rim, ephaptic centre and half-way between the two. Values of (D_{Na}, D_{Cl}) varied through (D_{Na}, D_{Cl}) = (0.65×10^9, 1.0×10^9) (a), (1.3×10^9, 2×10^9) (b) and (2.6×10^9, 4×10^9) $nm^2.s^{-1}$ (c) respectively.

D. Computational MATLAB coding

SPECIFY PARAMETERS

Geometry

```
% Ephaptic Radius (nm)
Disc_Radius = 8000;
% Axial Distance (nm)
Disc_Width = 20;
% Maximum Mesh Tetrahedral Length (nm)
Mesh_Hmax = 800;
```

Boundary Conditions

```
F1_Flux = [0,0];
F2_Flux = [0, 0];
```

Constants

```
% Diffusion Coefficients (nm2/s)
D_positive = 1.3e9;
D_negative = 2e9;
% Faraday's (C/mol)
Faraday = 96485.309;
% Elementary Charge (C)
Elementary_Charge = 1.60217733e-19;
% Gas Constant (J/K/mol)
Gas_Constant = 8.314511;
% Absolute Temperature (K)
Abs_Temp_Celcius = 37;
Abs_Temp_Kelvin = 273.15 + Abs_Temp_Celcius;
% Permittivity (F/nm)
permittivity_free_space = 8.854187817e-21;
permittivity_cytoplasm = 74.3e-9;
%Avogadro Constant (/mol)
Avogadro = 6.0221367e23;
%Specific Capacitance (F/nm2)
Sp_Capacitance = 1e-20;
%Moles of Na Extruded in Moles
Na_Extruded = (0.01*pi*(Disc_Radius/1e9)^2)*130e-3/Elementary_Charge/Avogadro;
%Calculation of starting Na
Starting_Na = 1.531e-25 - (Na_Extruded/(pi*Disc_Radius^2*Disc_Width));
% Generating Global variables for the coefficients of our PDEs which vary
% depending on current state of the solution
global a_coefficients
a_coefficients = Faraday / (Gas_Constant*Abs_Temp_Kelvin);
global omega_coefficients
omega_coefficients = Elementary_Charge / (permittivity_free_space*permittivity_cytoplasm);
global f_coefficients
f_coefficients =
(Faraday*Faraday*Disc_Width)/(Gas_Constant*Abs_Temp_Kelvin*2*Sp_Capacitance);
global D_pos;
D_pos = D_positive;
global D_neg;
D_neg = D_negative;
```

Timings

```
% Modelling End Time (s)
```

```
T_End = 80e-3;
% Number of Time Points to Sample
T_Resolution = 8000;
% Calculate Sampling Interval
T_Spacing = T_End / T_Resolution;
```

MODEL PROGRAMMING

Geometry and Mesh

```
% Produce a cylindrical geometry with dimensions specified above
gm = multicylinder(Disc_Radius,Disc_Width);
% Display geometry as assigned to PDE Model
model = createpde(2);
model.Geometry = gm;
% Figure display options
figure1 = figure;
pdegplot(model,'FaceAlpha',0.5,'FaceLabels',"on");
% Generate a mesh on the geometry with properties described above
meshgeom = generateMesh(model,"Hmax",Mesh_Hmax);
% Copy a component of matrix meshgeom to noderet for figure production
% later
noderet=meshgeom.Nodes;
% Figure display of meshed geometry
figure2 = figure;
pdeplot3D(model,"ElementLabels","off");
```

Coefficients

```
%COEFFICIENT C = Diffusion Coefficient for each of negative and positive
%ions
Model_Coefficient_c = [D_negative;D_positive];
% Specifying Coefficients
specifyCoefficients(model,"c",Model_Coefficient_c,"f",@fcoeffunction,"a",@acoeffunction,"d
",1,"m",0);
```

Boundary and Initial Conditions

```
% Apply a 0 flux across the Active membrane
applyBoundaryCondition(model,"neumann","Face",1,"g",F1_Flux,"q",0);
% Apply a flux across the Passive membrane equal to that specified in initial
% conditions
applyBoundaryCondition(model,"neumann","Face",2,"g",F2_Flux,"q",0);
% Apply a flux across the ephaptic rim - Either Neumann conditions for the
% action potential upstroke model or Dirichlet for the recovery model
%applyBoundaryCondition(model,"neumann","Face",3,"g",F3_Flux,"q",0);
applyBoundaryCondition(model,"dirichlet","Face",3,"u",[1.458e-25;1.531e-25]);
% Set initial PDE conditions - First option is resting conditions for modelling the action
potential upstroke, second option is the conditions at the end of the action potential
upstroke
u0 = [1.458e-25;Starting_Na];
setInitialConditions(model,u0);
%setInitialConditions(model,solution);
```

SOLUTION

Run model

```
%tlist is the initial time : Sampling Interval : end time in seconds
```

```
tlist = 0:T_Spacing:T_End;
solution = solvepde(model,tlist);
%Convert Nodal solution into uM from mol/nm3, saved as sol
sol_row_one = solution.NodalSolution(:,1,:)*1e30;
sol_row_two = solution.NodalSolution(:,2,:)*1e30;
Sol_time = solution.NodalSolution(:,:,:)*1e30;
```

DATA PRESENTATION

Heat Maps

```
% Display Chloride heat maps for the solution using PDE Toolbox
figure3 = figure;
pdeplot3D(model,"ColorMapData",sol_row_one(:,end)/1000,"Mesh","on");
```

```
figure4 = figure;
pdeplot3D(model,"ColorMapData",Sol_time(:,1,1)/1000,"Mesh","on");
```

```
% Display Sodium heat maps for the solution using PDE Toolbox
figure5 = figure;
pdeplot3D(model,"ColorMapData",sol_row_two(:,end)/1000,"Mesh","on");


figure6 = figure;
pdeplot3D(model,"ColorMapData",Sol_time(:,2,1)/1000,"Mesh","on");
```

```
% Display Ionic Concentration Difference heat map for the solution using PDE Toolbox
figure7 = figure;
pdeplot3D(model,"ColorMapData",(1.531e5-sol_row_two(:,end)),"Mesh","on");
```

```
% Produce a Membrane Potential Calibration Colour Bar for the solution using PDE Toolbox
figure8 = figure;
pdeplot3D(model,"ColorMapData",(((Faraday*(1.531e5-sol_row_two(:,end))*1e-3*Disc_Width*1e-
9)/2e-2)*1e6),"Mesh","on");
```

Time-Dependent Recovery Graphs

```
% Co-ordinates of points to sample [x,y,z]
nidcoords1 = [Disc_Radius,0,0.5*Disc_Width];
nidcoords2 = [0.5*Disc_Radius,0,0.5*Disc_Width];
nidcoords3 = [0,0,0.5*Disc_Width];
Rest_Cond = (tlist*0)+1.531e5;
% 3D Obtain Node function
getClosestNode = @(p,x,y,z) min((p(1,:) - x).^2 + (p(2,:) - y).^2 + (p(3,:) - z).^2);
% Assigns the closest node to the data points x,y,z to variable 'nid'
[~,nid1]=getClosestNode(meshgeom.Nodes,nidcoords1(1),nidcoords1(2),nidcoords1(3));
[~,nid2]=getClosestNode(meshgeom.Nodes,nidcoords2(1),nidcoords2(2),nidcoords2(3));
[~,nid3]=getClosestNode(meshgeom.Nodes,nidcoords3(1),nidcoords3(2),nidcoords3(3));
```

```
% Display a graph illustrating the recovery of sodium concentrations and
% the re-polarisation of the passive membrane with time
figure9 = figure;
yyaxis right
```

```
plot(tlist*1000,(((Faraday*(1.531e5-sol_row_two(nid1,:))*1e-3*Disc_Width*1e-9)/2e-
2)*1e6/1000),'k',"LineWidth",2.5,'LineStyle',"-");
yyaxis left
plot(tlist*1000,sol_row_two(nid1,:)/1000,'k',"LineWidth",2.5,'LineStyle',"--");
```

```
% Dispaly a graph illustrating the recovery of ionic concentration differences and
% the re-polarisation of the passive membrane with time
figure10 = figure;
yyaxis right
plot(tlist*1000,(((Faraday*(1.531e5-sol_row_two(nid1,:))*1e-3*Disc_Width*1e-9)/2e-
2)*1e6),'k',"LineWidth",2.5,'LineStyle',"--");
```

Spatio-Temporal Recovery Graph

```
% Create a grid of Values
xradial = -Disc_Radius:1:Disc_Radius;
yradial = 0*ones(1,length(xradial));
zradial = yradial;
```

```
%Interpolate the solution at each time point for the graph of
%spatio-temporal ephaptic recovery across the solution grid
interpolate_radialplot1 =
interpolateSolution(solution,xradial,yradial,zradial,[2],17)*1e30;
interpolate_radialplot2 =
interpolateSolution(solution,xradial,yradial,zradial,[2],32)*1e30;
interpolate_radialplot3 =
interpolateSolution(solution,xradial,yradial,zradial,[2],63)*1e30;
interpolate_radialplot4 =
interpolateSolution(solution,xradial,yradial,zradial,[2],126)*1e30;
interpolate_radialplot5 =
interpolateSolution(solution,xradial,yradial,zradial,[2],251)*1e30;
interpolate_radialplot6 =
interpolateSolution(solution,xradial,yradial,zradial,[2],501)*1e30;
interpolate_radialplot7 =
interpolateSolution(solution,xradial,yradial,zradial,[2],1001)*1e30;
interpolate_radialplot8 =
interpolateSolution(solution,xradial,yradial,zradial,[2],2001)*1e30;
interpolate_radialplot9 =
interpolateSolution(solution,xradial,yradial,zradial,[2],4001)*1e30;
interpolate_radialplot10 =
interpolateSolution(solution,xradial,yradial,zradial,[2],8001)*1e30;
```

```
% Produce figures with this new interpolation
figure11 = figure;
%Plot Membrane Potential Changes at the Passive Membrane
yyaxis right
plot(xradial,(((Faraday*(1.531e5-interpolate_radialplot1(:,1))*1e-3*Disc_Width*1e-9)/2e-
2)*1e6),'Color','k',"LineWidth",2.5,"LineStyle","--",'Marker','none');
hold on
plot(xradial,(((Faraday*(1.531e5-interpolate_radialplot2(:,1))*1e-3*Disc_Width*1e-9)/2e-
2)*1e6),'Color','#D95319',"LineWidth",2.5,"LineStyle","--",'Marker','none');
plot(xradial,(((Faraday*(1.531e5-interpolate_radialplot3(:,1))*1e-3*Disc_Width*1e-9)/2e-
2)*1e6),'Color','#7E2F8E',"LineWidth",2.5,"LineStyle","--",'Marker','none');
```

```
plot(xradial,(((Faraday*(1.531e5-interpolate_radialplot4(:,1))*1e-3*Disc_Width*1e-9)/2e-2)*1e6),'Color','#A2142F',"LineWidth",2.5,"LineStyle","--",'Marker','none');
plot(xradial,(((Faraday*(1.531e5-interpolate_radialplot5(:,1))*1e-3*Disc_Width*1e-9)/2e-2)*1e6),'Color','g',"LineWidth",2.5,"LineStyle","--",'Marker','none');
plot(xradial,(((Faraday*(1.531e5-interpolate_radialplot6(:,1))*1e-3*Disc_Width*1e-9)/2e-2)*1e6),'Color','b',"LineWidth",2.5,"LineStyle","--",'Marker','none');
plot(xradial,(((Faraday*(1.531e5-interpolate_radialplot7(:,1))*1e-3*Disc_Width*1e-9)/2e-2)*1e6),'Color','c',"LineWidth",2.5,"LineStyle","--",'Marker','none');
plot(xradial,(((Faraday*(1.531e5-interpolate_radialplot8(:,1))*1e-3*Disc_Width*1e-9)/2e-2)*1e6),'Color','m',"LineWidth",2.5,"LineStyle","--",'Marker','none');
plot(xradial,(((Faraday*(1.531e5-interpolate_radialplot9(:,1))*1e-3*Disc_Width*1e-9)/2e-2)*1e6),'Color','y',"LineWidth",2.5,"LineStyle","--",'Marker','none');
plot(xradial,(((Faraday*(1.531e5-interpolate_radialplot10(:,1))*1e-3*Disc_Width*1e-9)/2e-2)*1e6),'Color','r',"LineWidth",2.5,"LineStyle","--",'Marker','none');
ylabel('Membrane Potential (\muV)')
ylim([0,((Faraday*700*1e-3*Disc_Width*1e-9)/2e-2)*1e6])
yticks([0,10000,20000,30000,40000,50000,60000])
yticklabels({'0','10000','20000','30000','40000','50000','60000'});
hold off
%Plot Ionic Concentration Differences
yyaxis left
plot(xradial,(1.531e5-interpolate_radialplot1(:,1)),'Color','k',"LineWidth",2.5,"LineStyle","-",'Marker','none');
hold on
plot(xradial,(1.531e5-interpolate_radialplot2(:,1)),'Color','#D95319',"LineWidth",2.5,"LineStyle","-",'Marker','none');
plot(xradial,(1.531e5-interpolate_radialplot3(:,1)),'Color','#7E2F8E',"LineWidth",2.5,"LineStyle","-",'Marker','none');
plot(xradial,(1.531e5-interpolate_radialplot4(:,1)),'Color','#A2142F',"LineWidth",2.5,"LineStyle","-",'Marker','none');
plot(xradial,(1.531e5-interpolate_radialplot5(:,1)),'Color','g',"LineWidth",2.5,"LineStyle","-",'Marker','none');
plot(xradial,(1.531e5-interpolate_radialplot6(:,1)),'Color','b',"LineWidth",2.5,"LineStyle","-",'Marker','none');
plot(xradial,(1.531e5-interpolate_radialplot7(:,1)),'Color','c',"LineWidth",2.5,"LineStyle","-",'Marker','none');
plot(xradial,(1.531e5-interpolate_radialplot8(:,1)),'Color','m',"LineWidth",2.5,"LineStyle","-",'Marker','none');
plot(xradial,(1.531e5-interpolate_radialplot9(:,1)),'Color','y',"LineWidth",2.5,"LineStyle","-",'Marker','none');
plot(xradial,(1.531e5-interpolate_radialplot10(:,1)),'Color','r',"LineWidth",2.5,"LineStyle","-",'Marker','none');
figure11.CurrentAxes.LineWidth = 3;
figure11.CurrentAxes.FontSize = 20;
figure11.CurrentAxes.XTick = [-Disc_Radius:2000:Disc_Radius];
hold off
```

Midline Slice Plot

```
Slice_Plot_Resolution = 10;
%Time Point for the midline-slice - Correspond to the three critical
%time points
T_For_Slice_Plot = T_Resolution + 1;
%T_For_Slice_Plot = 1001;
%T_For_Slice_Plot = 126;
[xslice,zslice]=meshgrid(-
Disc_Radius:Slice_Plot_Resolution:Disc_Radius,linspace(0,Disc_Width,((1/Slice_Plot_Resolut
ion)*2*100+1)));
yslice = 0*ones(size(xslice));
interpolate_radialplot11 =
interpolateSolution(solution,xslice,yslice,zslice,[1,2],T_For_Slice_Plot)*1e30;
interpolate_radialplot12 = reshape(interpolate_radialplot11(:,1),size(xslice));
interpolate_radialplot13 = reshape(interpolate_radialplot11(:,2),size(xslice));
```

```
figure12 = figure;
imagesc(interpolate_radialplot12/1000,"XData",xslice(1,:),'YData',zslice(:,1));
```

```
%Display Midline Slice Plot for Sodium Ions
figure13 = figure;
imagesc(interpolate_radialplot13/1000,"XData",xslice(1,:),'YData',zslice(:,1));
```

```
%Display Midline Slice Plot for Ionic Concentration Differences
figure14 = figure;
imagesc((1.531e5-interpolate_radialplot13),"XData",xslice(1,:),'YData',zslice(:,1));
```

```
%Produce a Calibration bar for membrane potential changes
figure15 = figure;
imagesc(((Faraday*(1.531e5-interpolate_radialplot13)*1e-3*Disc_Width*1e-9)/2e-
2)*1e6,"XData",xslice(1,:),'YData',zslice(:,1));
```

Midline Slice Plots with Times

```
%Produce a Midline slice plot that shows successive slice in time
Slice_Plot_Resolution = 1;
T_For_Slice_Plot = [T_Resolution+1, round((T_Resolution+1)/2), round((T_Resolution+1)/4),
round((T_Resolution+1)/8), round((T_Resolution+1)/16), round((T_Resolution+1)/32),
round((T_Resolution+1)/64), round((T_Resolution+1)/128),
round((T_Resolution+1)/256),round((T_Resolution+1)/512)];
[xslice1,zslice1]=meshgrid(-
Disc_Radius:Slice_Plot_Resolution:Disc_Radius,1,((1/Slice_Plot_Resolution)*2*30+1));
yslice1 = 0*ones(size(xslice1));
zslice1 = 0*ones(size(xslice1));
for i = [1:10]
   my_field = strcat('v',num2str(i));
   interpolation.(my_field) =
interpolateSolution(solution,xslice1,yslice1,zslice1,[1,2],T_For_Slice_Plot(i))*1e30;
   interpolationreshaped.(my_field) =
(interpolateSolution(solution,xslice1,yslice1,zslice1,[1,2],T_For_Slice_Plot(i))*1e30);
end
matrixtoplotna =
[interpolationreshaped.v1(:,2),interpolationreshaped.v2(:,2),interpolationreshaped.v3(:,2)
,interpolationreshaped.v4(:,2),interpolationreshaped.v5(:,2),interpolationreshaped.v6(:,2)
,interpolationreshaped.v7(:,2),interpolationreshaped.v8(:,2),interpolationreshaped.v9(:,2)
,interpolationreshaped.v10(:,2)];
```

```
matrixtoplotna = fliplr(matrixtoplotna);
matrixtoplotcl =
[interpolationreshaped.v1(:,1),interpolationreshaped.v2(:,1),interpolationreshaped.v3(:,1)
,interpolationreshaped.v4(:,1),interpolationreshaped.v5(:,1),interpolationreshaped.v6(:,1)
,interpolationreshaped.v7(:,1),interpolationreshaped.v8(:,1),interpolationreshaped.v9(:,1)
,interpolationreshaped.v10(:,1)];
matrixtoplotcl = fliplr(matrixtoplotcl);
```

```
%Display Midline Slice plot with successive slices in time for Sodium
figure16=figure;
imagesc(matrixtoplotna'/1000,"XData",xslice1(1,:),'YData',zslice1(:,1));
```

```
%Display Midline Slice plot with successive slices in time for Ionic
%Concentration Differences
figure17=figure;
imagesc((1.531e5-(matrixtoplotna')),"XData",xslice1(1,:),'YData',zslice1(:,1));
```

```
%Produce a Calibration Bar for Membrane Potential Changes
figure18=figure;
imagesc(((Faraday*(1.531e5-(matrixtoplotna'))*1e-3*Disc_Width*1e-9)/2e-
2)*1e3*1000,"XData",xslice1(1,:),'YData',zslice1(:,1));
```

```
%Display Midline Slice plot with successive slices in time for chloride
figure19=figure;
imagesc(matrixtoplotcl'/1000,"XData",xslice1(1,:),'YData',zslice1(:,1));


%Determination of the a coefficient for the PDEs as they vary with the
%state of the current solution

function a = acoeffunction(region, state)
global a_coefficients
global omega_coefficients
global D_neg
global D_pos
N_char = 2;
nr = numel(region.y);
a = zeros(N_char,nr);
a(1,:) = D_neg *a_coefficients * omega_coefficients * (state.u(1)-state.u(2));
a(2,:) = -1 * D_pos * a_coefficients * omega_coefficients * (state.u(1)-state.u(2));
end
function f = fcoeffunction(region, state)
global f_coefficients
global D_neg
global D_pos
NC = 2;
Uone = gradient(state.u(1));
Utwo = gradient(state.u(2));
f = zeros(NC, length (region.x));
f(1,:) = -1 * D_neg * f_coefficients*dot((Utwo-Uone),Uone);
f(2,:) = D_pos * f_coefficients*dot((Utwo-Uone),Utwo);
end
```
